# Interleukin-22 suppresses major histocompatibility complex II in mucosal epithelial cells

**DOI:** 10.1084/jem.20230106

**Published:** 2023-09-11

**Authors:** Md Moniruzzaman, M. Arifur Rahman, Ran Wang, Kuan Yau Wong, Alice C.-H. Chen, Alexandra Mueller, Steven Taylor, Alexa Harding, Thishan Illankoon, Percival Wiid, Haressh Sajiir, Veronika Schreiber, Lucy D. Burr, Michael A. McGuckin, Simon Phipps, Sumaira Z. Hasnain

**Affiliations:** 1 Faculty of Medicine, The University of Queensland, Brisbane, Australia; 2 Immunopathology Group, Translational Research Institute, Mater Research Institute—The University of Queensland, Brisbane, Australia; 3 South Australian Health and Medical Research Institute, Adelaide, Australia; 4 Faculty of Medicine, Dentistry and Health Sciences, The University of Melbourne, Parkville, Australia; 5 Australian Infectious Diseases Research Centre, The University of Queensland, Brisbane, Australia; 6 Department of Respiratory and Sleep Medicine, Mater Health, South Brisbane, Australia; 7 Respiratory Immunology Laboratory, QIMR Berghofer Medical Research Institute, Herston, Australia

## Abstract

Major histocompatibility complex (MHC) II is dynamically expressed on mucosal epithelial cells and is induced in response to inflammation and parasitic infections, upon exposure to microbiota, and is increased in chronic inflammatory diseases. However, the regulation of epithelial cell–specific MHC II during homeostasis is yet to be explored. We discovered a novel role for IL-22 in suppressing epithelial cell MHC II partially via the regulation of endoplasmic reticulum (ER) stress, using animals lacking the interleukin-22-receptor (IL-22RA1), primary human and murine intestinal and respiratory organoids, and murine models of respiratory virus infection or with intestinal epithelial cell defects. IL-22 directly downregulated interferon-γ–induced MHC II on primary epithelial cells by modulating the expression of MHC II antigen A α (H2-Aα) and Class II transactivator (Ciita), a master regulator of MHC II gene expression. IL-22RA1-knockouts have significantly higher MHC II expression on mucosal epithelial cells. Thus, while IL-22–based therapeutics improve pathology in chronic disease, their use may increase susceptibility to viral infections.

## Teaser

IL-22 directly suppresses interferon-γ–induced intestinal and respiratory epithelial cell MHC II. While suppression of IL-22–driven epithelial MHC II may be beneficial in chronic inflammatory conditions, it may increase susceptibility to infection by delaying initiation of appropriate immune responses.

## Graphical Abstract

**Figure:**
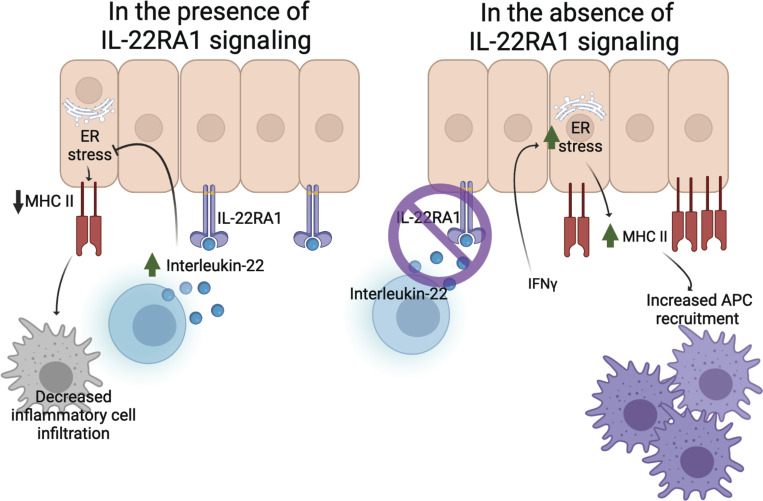

## Introduction

Mucosal epithelial cells can modulate immune responses through the secretion of cytokines, chemokines, and antimicrobials, the transfer of antibodies to the apical surface, and the transportation of antigens to professional antigen-presenting cells (APCs) in the underlying mucosa. These APCs, such as dendritic cells and macrophages, constitutively express high levels of MHC II, which presents antigens to CD4^+^ T cells to promote tolerance or initiate an immune response. However, epithelial cells can also express MHC II, which is induced in response to inflammation and is increased in inflammatory bowel disease, parasitic infections, and upon the introduction of bacteria in germ-free animals (Hershberg et al., 1997, 1998; Koyama et al., 2019; Smillie et al., 2019). Recent work highlighted that stem cell–like intestinal epithelial cells (IECs) express MHC II, which may help in activation of intestine-resident immune cells and/or maintain epithelial cell differentiation (Biton et al., 2018; Sanderson et al., 2004; Westendorf et al., 2009). Despite these observations, epithelial MHC II expression and function remain elusive, and how epithelial MHC II is regulated during homeostasis is largely unknown.

Several studies have shown that T helper cell cytokines, such as IFNγ, can upregulate epithelial MHC II (Sanderson et al., 2004). While IL-10 has been shown to downregulate MHC II on professional APCs (Koppelman et al., 1997; Mittal and Roche, 2015), no reports have described what controls epithelial MHC II. The IL-22 receptor, IL-22RA1, is highly expressed in mucosal epithelial cells, and IL-22 is a critical regulator of homeostasis (Hasnain and Begun, 2019). We have previously shown that IL-22 restores IEC integrity and shifts in colonic microbiota in high-fat diet–induced obese mice through the direct control of epithelial endoplasmic reticulum (ER) stress and subsequent inflammation (Gulhane et al., 2016). As the expression of IL-22RA1 is limited to epithelial cells, the anti-inflammatory effects of IL-22 are considered to be indirect (Gurney, 2004; Wolk et al., 2004); however, the mechanisms that confer this immunosuppressive function remain largely unknown. Indirect stimulation of IL-22RA1 through IL-22 gene transduction or IL-22–producing immune cell transfer has shown beneficial impacts in small intestinal epithelial regeneration (Lindemans et al., 2015) and in dextran sodium sulfate (DSS)–induced ulcerative colitis (Sugimoto et al., 2008; Zenewicz et al., 2008). Different forms of IL-22 are progressing to clinical trials for inflammatory bowel disease (IBD) and have demonstrated significant anti-inflammatory effects (Zheng et al., 2008).

Here, using intestinal and respiratory primary organoid assays, we highlight a new and direct functional anti-inflammatory role for IL-22 by suppressing MHC II on epithelial cells. We characterized the functional impact of IL-22–mediated MHC II suppression by using mice that lack IL-22 receptor signaling. We showed that while IL-22–mediated suppression of epithelial cell–MHC II is beneficial in a chronic inflammatory setting, during acute inflammation the same phenomenon can be detrimental. Our work provides an explanation for the contrasting reports of pro- and anti-inflammatory roles of IL-22.

## Results and discussion

### Epithelial MHC II is driven by inflammation

**Figure 1.**
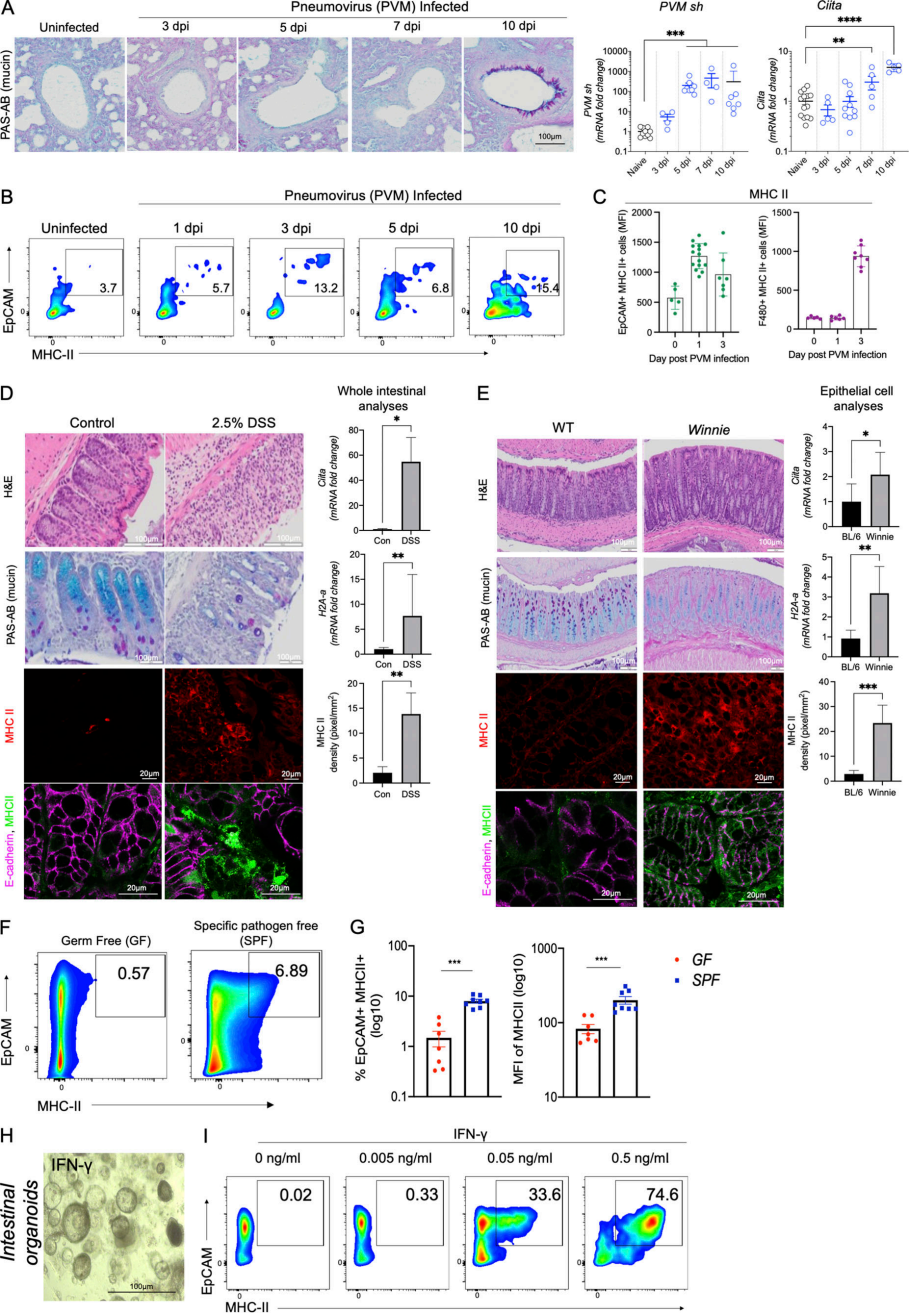
**Upregulation of MHC II on mucosal epithelial cells during infection and inflammation. (A)** Representative images of respiratory tract histology highlighting mucus-secreting goblet cells (PAS-AB staining) in C57BL/6 animals infected with PVM (10 PFU). PVM small hairpin protein (*PVM-sh*) and *Ciita* (MHC II transactivator) mRNA expression levels in whole respiratory tract were assessed by qRT-PCR at indicated dpi. **(B)** Representative flow cytometric plots. **(C)** Total MFI of MHC II expressing respiratory tract Epcam^+ve^ and F4/80^+ve^ cells in PVM infected animals on 0, 1, and 3 dpi. **(D)** Representative colon pathology (H&E), mucin-producing goblet cells (PAS-AB), and MHC II, MHC II/E-cadherin in the intestines of WT mice challenged with DSS (2.5% in drinking water) for 7 d. Colonic mRNA expression of *Ciita* and *H2A-α*, and quantitation of epithelial MHC II (pixel/mm^2^) in WT and DSS-treated animals. **(E)** Representative colon pathology (H&E), mucin-producing goblet cells (PAS-AB), and MHC II, MHC II/E-cadherin in the intestines of WT mice and Winnie animals with emerging colitis (6–8 wk of age). mRNA expression levels of *Ciita* and *H2A-a* from IECs isolated and quantitation of epithelial MHC II (pixel/mm^2^) from WT and *Winnie* animals. **(F and G)** (F) Representative flow cytometric plots, relative frequency, and (G) MFI of colonic MHC II^+ve^ epithelial cells harvested from GF and SPF C57BL/6 mice. **(H and I)** Intestinal organoids were treated with IFNγ (24 h) with increasing doses and total MHC II assessed using flow cytometry. Statistics: mean ± SEM (*n* = 4–14); data are representative of two independent experiments. **(A and B)** One-way ANOVA, Bonferroni’s post hoc test. **(C and D)** *t* test; *P < 0.05, **P < 0.01, ***P < 0.001, and ****P < 0.0001.

**Figure S1.**
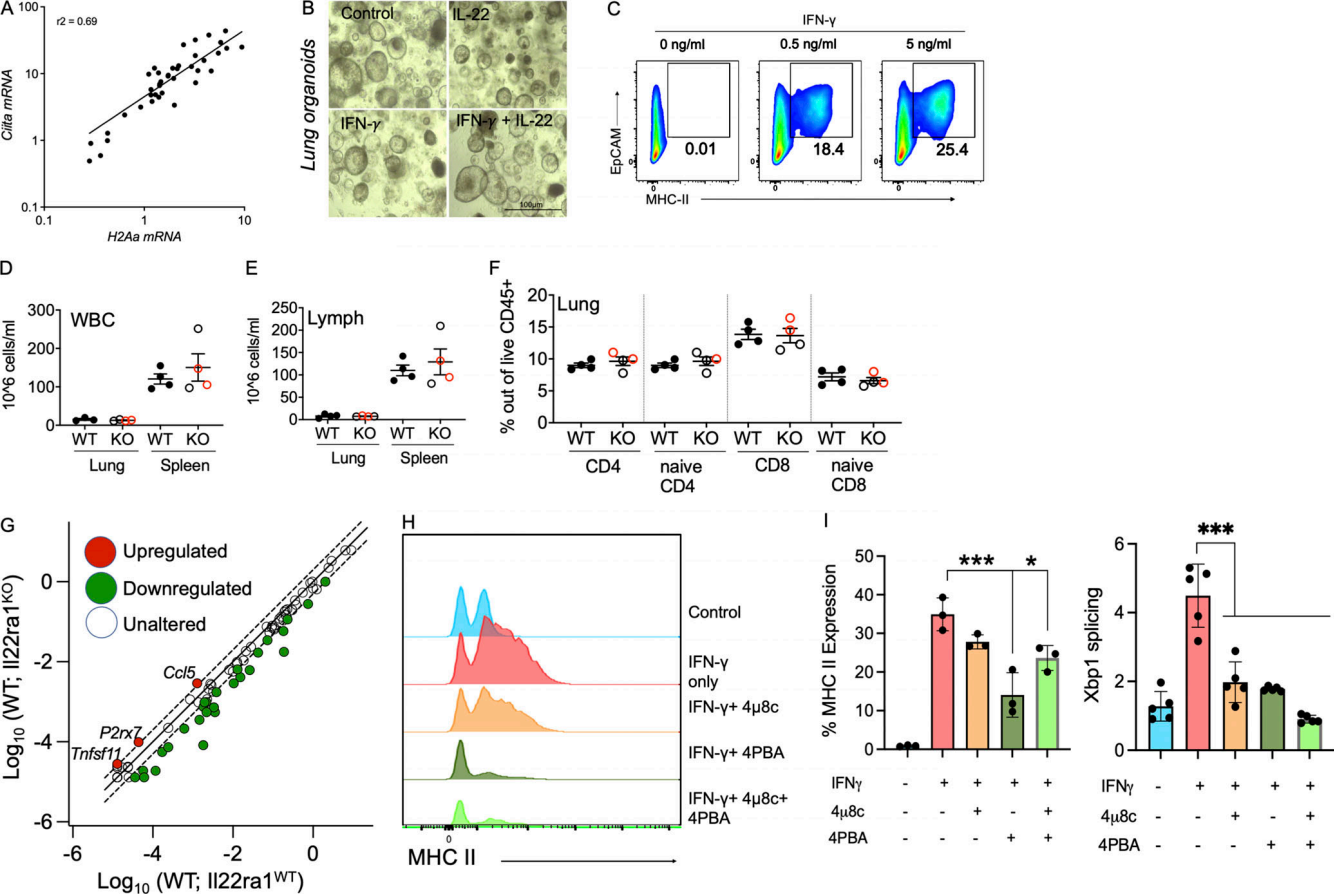
**Inhibition of ER stress partially reduced IFNγ-induced epithelial MHC II. (A)** Pearson correlation between log-transformed H2A-a and *Ciita* gene expression in the lung of PVM-infected C57BL/6 mice. **(B)** Representative microscopic images of lung organoids treated with IFNγ (5 ng/ml) alone (shown in Fig. 1 H) or in combination with IL-22 (10 ng/ml). **(C)** Lung organoids were treated with IFNγ in a dose-dependent manner and MHC II assessed using flow cytometry; representative flow cytometric plots shown here. **(D and E)** Total count of white blood cells (WBC; D) and (E) differential count of lymphocytes measured by Mindray assay in the lung and spleen of 6–8-wk-old Il22ra1 fl/fl (Il22ra1^WT^) and CMV-cre × Il-22ra1 fl/fl (Il22ra1^KO^) mice. **(F)** Flow cytometric relative frequencies of CD4 and CD8 T cells in the lungs isolated from *Il22ra1*^*WT*^ and *Il22ra1*^*KO*^ mice as in B. Red = female; black = male. **(G)** Colon from *Il22ra1*^*WT*^ and *Il22ra1*^*KO*^ mice was analyzed using cytokines and chemokines RT^2^ Profiler PCR Array. Genes upregulated (red) are annotated. **(H and I)** Flow cytometry histograms for total MHC II in 16HBEs treated with IFNγ alone or in combination with ER stress inhibitors (4PBA and 4 4µ8c; H) with % MHC II expression (I). **(J)** mRNA levels of splicing of Xbp1 confirming inhibition with inhibitors. Data are presented as mean ± SEM (*n* = 3–4). Data are representative of two independent experiments and are presented as mean ± SEM (*n* = 5–8). One-way ANOVA, Bonferroni’s post hoc test; *P < 0.05 and ***P < 0.001.

Epithelial cells were shown to express MHC II molecules in 1980; however, most of the focus at the mucosa has been on microfold cells as antigen presenters (Heuberger et al., 2021). While microfold cells are localized to the Peyer’s patches and are rare, epithelial cells line the whole mucosa and respond to inflammation. To confirm the upregulation of epithelial MHC II during acute and chronic inflammation, we utilized mouse models of respiratory infection and intestinal inflammation. Using the model of murine pneumovirus infection (Domachowske et al., 2001; Sikder et al., 2023), we demonstrated that the progressive increase in viral load in epithelial cells is followed by epithelial pathology, including goblet cell hyperplasia as shown by period-acid Schiffs-Alcian Blue (PAS-AB) staining; Fig. 1 A) and an increase in Epcam^+ve^ epithelial MHC II expression (Fig. 1 B). Class II transactivator, Ciita, is a transcriptional master regulator of MHC II genes, and increased *Ciita* expression significantly correlated with *H2A-a* (histocompatibility 2, class II antigen A) mRNA (Fig. 1 A and Fig. S1 A). Mean fluorescence intensity (MFI) demonstrated that the increase in MHC II on epithelial cells occurred prior to the increase in MHC II on macrophages in the lung (Fig. 1 C). Intestinal epithelial MHC II is increased in IBD (Fritz et al., 2011; Hepworth et al., 2015). Using the chemical-induced DSS model of colitis, we confirmed that, along with increased pathology, mRNA expression of MHC II (*H2A-a*) and *Ciita* were increased (Fig. 1 D). However, in this model, there was significant DSS-induced epithelial damage, as can be seen in the H&E-stained tissue, and so the increased expression of these genes could be due to recruitment of macrophages or other MHC II expressing leukocytes (Fig. 1 D). Therefore, we utilized a spontaneous model of colitis that develops in *Winnie* mice, which carry a *Muc2* gene mutation resulting in an epithelial cell defect and inflammation (Fig. 1 E; Hasnain et al., 2013; Wang et al., 2021). IEC were isolated from the *Winnie* and wild-type (WT) animals and revealed an increase in *H2A-a* and *Ciita* in *Winnie* epithelial cells. This was also mirrored by the MHC II increase in E-cadherin–positive epithelial cells (Fig. 1 E). Overall, these findings confirm that mucosal epithelial cells in the respiratory tract and intestine can upregulate MHC II in response to inflammation (Wosen et al., 2018).

To further investigate the changes in epithelial cells in a controlled manner, we used the organoid culture system, which was passaged at least four to five times to ensure the absence of immune cells. IECs have been shown to lose MHC II expression after prolonged culturing (Biton et al., 2018), which could be due to the absence of microbial molecules and/or the host cytokine microenvironment (Koyama et al., 2019). We confirmed low epithelial MHC II in small intestinal epithelial cells from germ-free (GF) animals compared with specific pathogen–free (SPF) animals (Fig. 1, F and G). After establishment of culture ex vivo, MHC II expression was negligible on both intestinal or respiratory organoids, as previously reported (Wosen et al., 2018; Fig. 1, H and I; Fig. S1, B and C). Consistent with previous reports, IFNγ increased MHC II on the Epcam^+ve^ intestinal and respiratory cells in a dose-dependent manner (Fig. 1 I and Fig. S1, B and C; Jamwal et al., 2020; Jurewicz and Stern, 2019; Koyama et al., 2019). However, the signals that suppress this pathway on epithelial cells have remained elusive.

### Ablation of IL-22**–**signaling expands epithelial cell**–**MHC II expression

**Figure 2.**
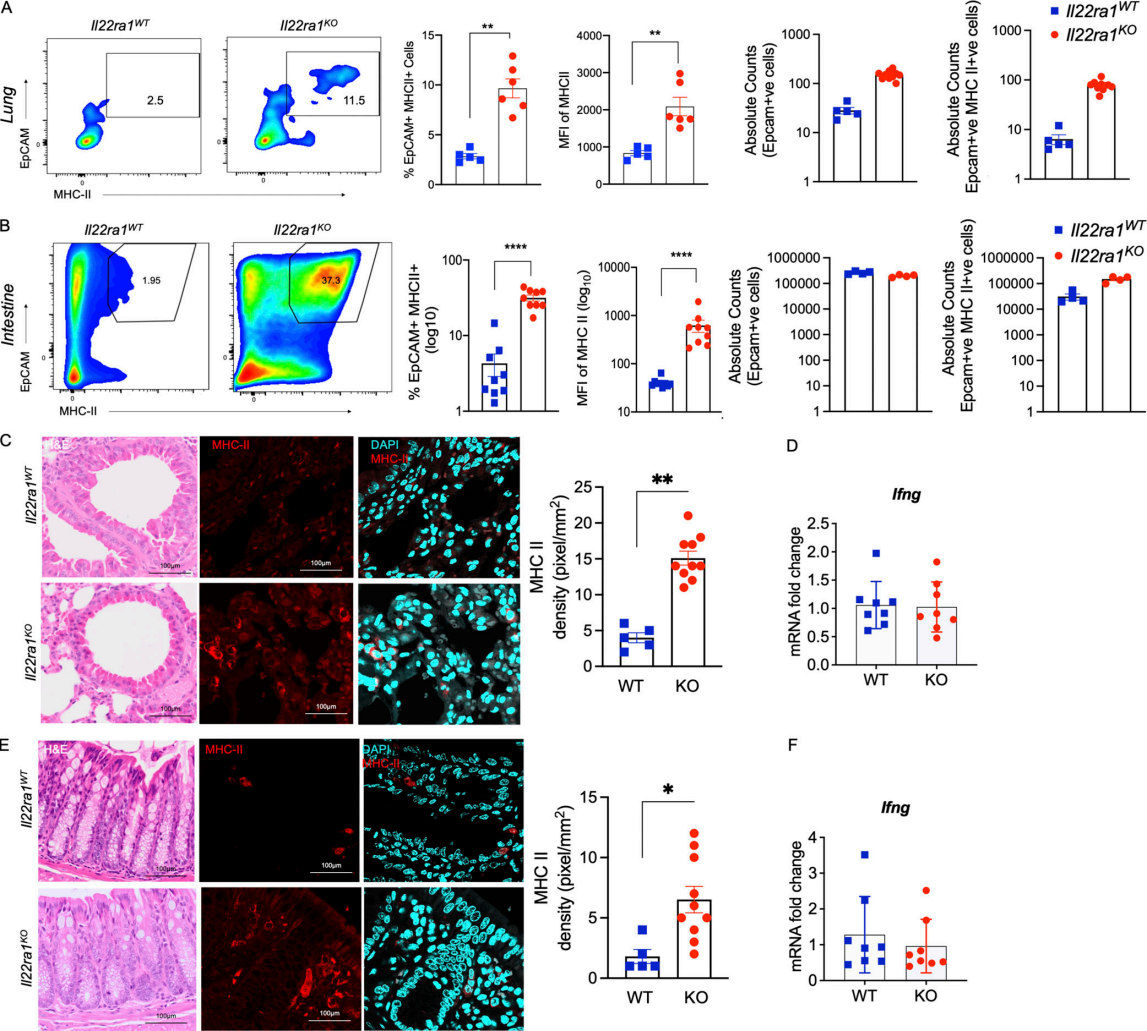
**Endogenous IL-22 suppresses epithelial MHC II. (A and B)** Flow cytometry plots of relative frequency, MFI of total MHC II expression, and absolute Epcam^+ve^ and Epcam^+ve^ MHCII^+ve^ counts in lung (A) and colonic (B) epithelial cells isolated from naïve Il22ra1 fl/fl (*Il22ra1*^*WT*^) and CMV-cre × Il-22ra1 fl/fl (*Il22ra1*^*KO*^) mice. **(C and E)** Representative pathology (H&E) and MHC II expression and quantitation (pixels/mm^2^) in the lungs (C) and intestine (E) of *Il22ra1*^*WT*^ *and Il22ra1*^*KO*^ animals. **(D and F)** mRNA levels of *Ifng* in the lung (D) and intestine (F) in the of *Il22ra1*^*WT*^ *and Il22ra1*^*KO*^ animals. Statistics: mean ± SEM (*n* = 5–9); data are representative of two independent experiments. *t* test; *P < 0.05, **P < 0.01, and ****P < 0.0001.

Mucosal epithelial cells are exposed to a range of antigens derived from the environment, food, and microbes, and are in close contact with immune cells, such as the alveolar macrophages in the lung and intraepithelial lymphocytes and underlying APCs in the gut. As non-professional APCs, epithelial cells have been reported to have the ability to process and present antigen via MHC II, and epithelial MHC II also contributes to epithelial cell renewal and differentiation (Biton et al., 2018; Heuberger et al., 2021). Nevertheless, uncontrolled overactivation of epithelial MHC II could promote autoimmunity; therefore, tight regulation is required. Interestingly, using flow cytometry and immunofluorescence staining, we observed a large increase in MHC II expression in Epcam^+ve^ cells in the absence of IL-22RA1 signaling in both the lung and intestine (Fig. 2, A–C and E). While no changes in the absolute numbers of Epcam^+ve^ MHC II^+ve^ cells were noted in the intestine, there were changes in the lungs. There was also a slight increase in MHC II expression in Epcam^−ve^ cells in the IL-22RA1-knockout mice (*Il22ra1*^*KO*^), which could be due to the endothelial cells (Fig. 2, A and B). Despite the increased epithelial MHC II expression, there was no effect on white blood cell count or lymphocytes (including CD4 and CD8 T cell compartments) in the lung or spleen between the *Il22ra1*^WT^ and *Il22ra1*^*KO*^ animals (Fig. S1, D–F). Importantly, RT^2^ PCR array profiler demonstrated minimal alterations in the *Il22ra1*^WT^ and *Il22ra1*^*KO*^ (Fig. S1 G), and no changes were observed in the *Ifng* levels in the intestine and lung (Fig. 2, D and F). This is consistent with several previous reports of *Il22ra1*^*KO*^ having no apparent phenotype without stimulus (Zheng et al., 2016).

We have previously shown that IL-10 and IL-22 can suppress ER stress in epithelial cells (Gulhane et al., 2016; Hasnain et al., 2013). However, exactly how ER stress affects processing of antigen and the efficiency of MHC II antigen presentation is unclear (Granados et al., 2009; Leung, 2015; Osorio et al., 2018). Nevertheless, inhibition of ER stress using 4μ8c (IRE1 inhibitor) and/or 4PBA (generic stress inhibitor) inhibited Xbp1 splicing induced by IFNγ and also inhibited the IFNγ-induced upregulation of MHC II in epithelial cells (Fig. S1, H and I).

Overall, our findings are consistent with previous reports that emphasized the importance of high local production of IL-22 at the epithelial barrier, including the lung and intestine, during homeostasis. Therefore, we speculate that the suppression of epithelial MHC II by IL-22 could be critical for tolerance to food and microbial antigens.

### IL-22**–**mediated suppression of epithelial MHC II is associated with reduced pathology in chronic inflammation

**Figure 3.**
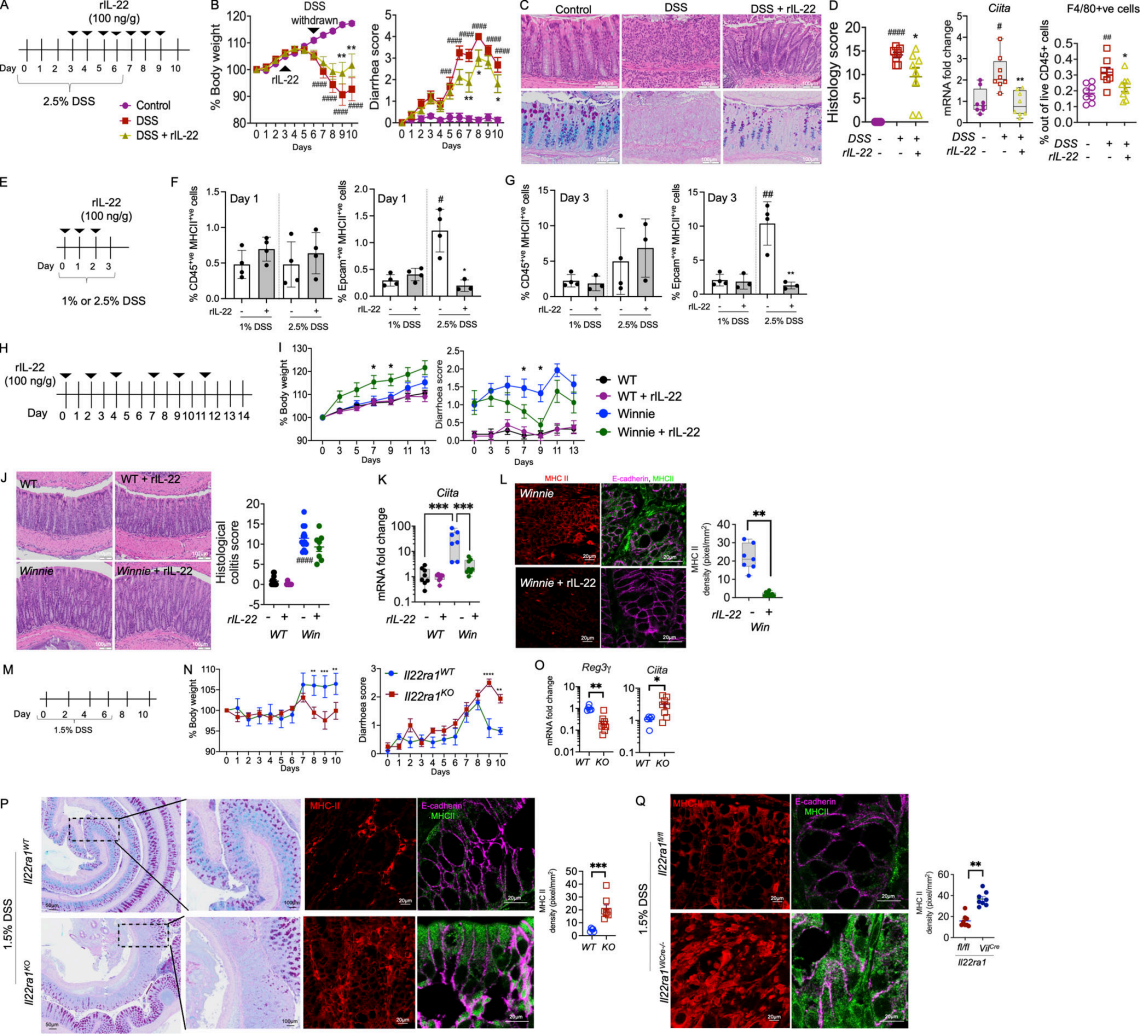
**IL-22–suppressed epithelial MHC II correlates with reduced pathology in chronic colonic inflammation. (A)** Schematic representation of the experimental plan; C57BL/6 mice were challenged with 2.5% DSS and a subgroup of animals were treated daily with rIL-22 (100 ng/g/body weight) starting from day 3 **(B)** Body weight (as % of starting weight) and diarrhea scores. **(C)** H&E and PAS-AB staining highlighting goblet cells. **(D)** Blind histological colitis score, *Ciita* mRNA expression in the intestine, and flow cytometric evaluation of the relative frequency of colonic F4/80^+^ macrophages. **(E–G)** Schematic representation of experimental plan (E); C57BL/6 mice were challenged with 1 or 2.5% DSS and MHC II assessed on immune (CD45^+ve^) or epithelial (Epcam^+ve^) cells using flow cytometry on day 1 (F) and day 3 (G). **(H)** Schematic diagram showing Winnie mice with emerging colitis treated with rIL-22 (100 ng/g/body weight) on alternative days for 2 wk prior to sacrifice. **(I)** Body weight loss and diarrhea score. **(J)** H&E staining (WT and Winnie same as shown in Fig. 1 E) and blind histological colitis score. **(K and L)** qRT-PCR showing *Ciita* mRNA expression (K) and (L) representative images and quantitation (pixels/mm^2^) of MHC II expression (with and without E-cadherin) in the intestine. **(M)** Schematic diagram showing DSS (1.5% in drinking water) challenge of *Il22ra*^*WT*^ (littermate controls) and *Il22ra1*^*KO*^ mice for 6 d with a 4-d recovery. **(N)** Body weight loss and diarrhea score. **(O)** Colonic mRNA expression levels of antimicrobial peptide *Reg3β* and *Ciita* measured by qRT-PCR. **(P)** PAS-AB and MHC II, MHC II/E-cadherin staining and quantitation in *Il22ra*^*WT*^ and *Il22ra1*^*KO*^ exposed to DSS. **(Q)** MHC II, MHC II/E-cadherin staining and quantitation in the intestine of *Il22ra*^*WT*^ and *Il22ra1*^*VilCre−/−*^ mice exposed to 1.5% DSS. Statistics: mean ± SEM (*n* = 6–12); data are representative of three independent experiments. One-way ANOVA, Bonferroni’s post hoc test; ^#^P < 0.05; ^##^P < 0.01; ^###^P < 0.001; ^####^P < 0.0001 compared with control and *P < 0.05; **P < 0.01; ***P < 0.001 compared with DSS group.

**Figure S2.**
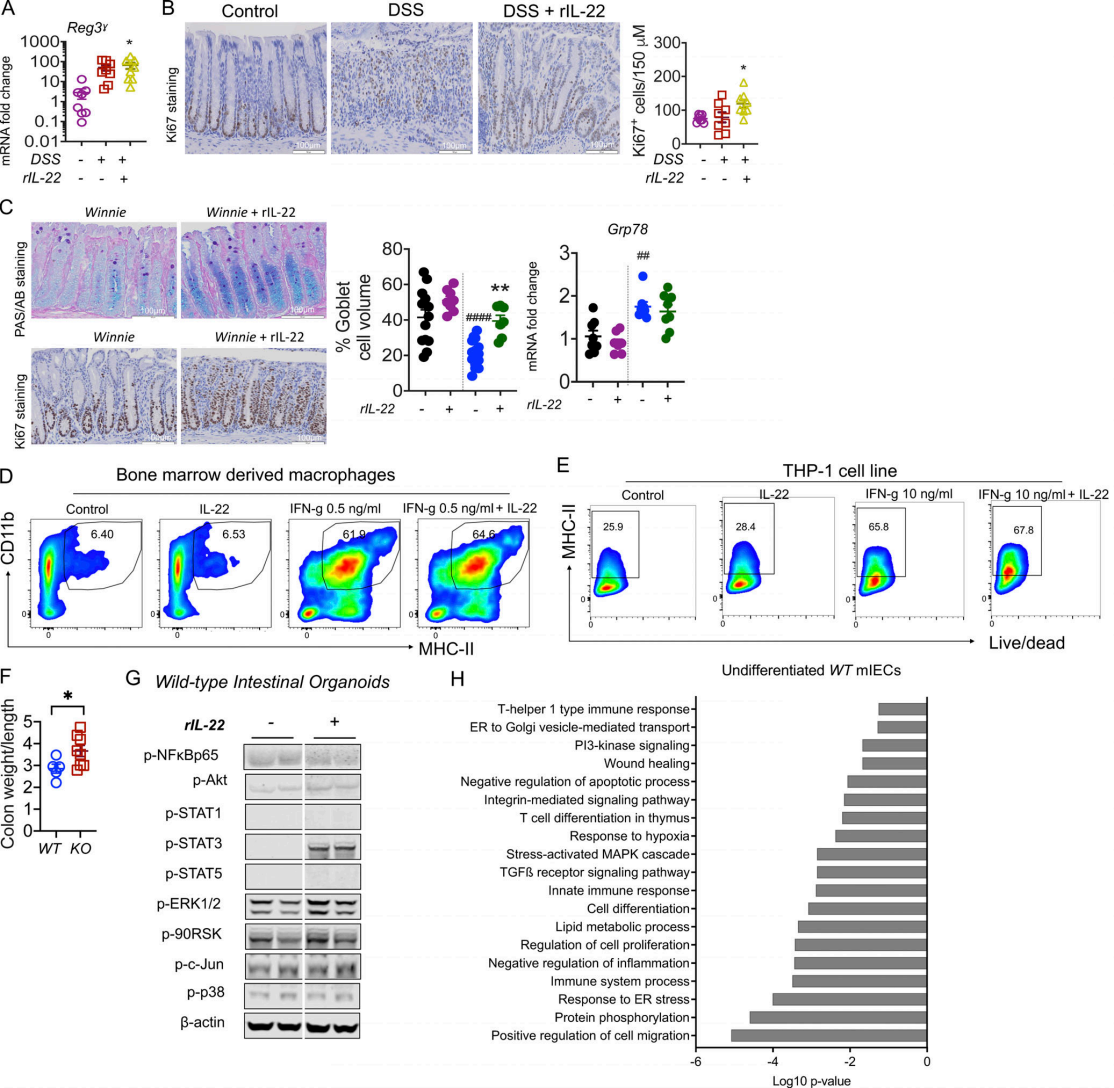
**IL-22 reduced pathology during intestinal inflammation. (A)** Gene expression level of antimicrobial peptide *Reg3*γ in the intestine on day 10 of the C57BL/6 mice challenged with 2.5% DSS for the first 6 d and treated with rIL-22 starting from day 3 to day 10. **(B)** Representative images of Ki67 staining and quantitative data showing Ki67^+ve^ staining increase with rIL-22 treatment in DSS mice. **(C)** Representative PAS-AB staining and Ki67 staining, blind histological scoring for goblet cell volume, and relative expression of ER stress marker *Grp78* in the distal colons of *Winnie* mice treated with or without rIL-22 on alternative days for 2 wk prior to cull at day 14. **(D)** Flow cytometric plots and data shown as a % of CD45^+^, Cd11b^+^ MHC II^+^ BMDMs from WT animals in the presence of IFNγ and IL-22 alone or in combination (*n* = 3). **(E)** Flow cytometric plots and MHC II^+ve^ cells shown as a percentage of live/dead THP1 cells treated with IFNγ and IL-22 alone or in combination. **(F)** Colon weight/length ratio of naïve Il22ra1 fl/fl (Il22ra1^WT^) and CMV-cre × Il-22ra1 fl/fl (Il22ra1^KO^) mice. **(G)** Cell signaling activation markers were measured by Western blot analysis in intestinal organoids from WT animals with and without IL-22 (rIL-22, 100 ng/ml for 30 min). **(H)** DAVID gene ontology analysis of RNA-Seq data shows a downregulation of pathways associated with ER stress, inflammation in WT mice treated with IL-22 (rIL-22, 100 ng/ml for 4 h). Data are representative of three independent experiments and are presented as mean ± SEM (*n* = 4–12). One-way ANOVA, Bonferroni’s post hoc test; *P < 0.05, **P < 0.01. ^##^P < 0.01, ^####^P < 0.0001 compared to untreated WT controls. Source data are available for this figure: SourceData FS2.

To provide further evidence that IL-22 can suppress epithelial MHC II expression, we used the chemical-induced colitis model. We administered DSS in drinking water for 6 d with 3 d for recovery (Fig. 3 A). Corroborating previous reports, daily administration of rIL-22 at 100 ng/g from day 3 improved body weight loss, diarrhea scores, and histological colitis, which was accompanied by an increase in proliferation (Fig. 3, B–D; and Fig. S2, A and B; Sugimoto et al., 2008). The significant reduction in DSS-induced *Ciita* in the colon was associated with reduced macrophage infiltration (Fig. 3, C and D). Early assessment of epithelial cell MHC II in animals given 1 or 2.5% DSS revealed that MHC II expression is elevated on Epcam^+ve^ cells prior to its increase in MHC II on immune cells (CD45^+ve^; Fig. 3, E–G). Similarly, in spontaneous *Winnie* colitis, rIL-22 improved body weight gain, significantly reduced diarrhea scores, and improved pathology, as evidenced by a reduction in histological scores, increased epithelial proliferation, and restoration of goblet cells in the intestine (PAS staining and goblet cell volume; Fig. 3, H–J; and Fig. S2 C). This was accompanied by a reduction in colonic *Ciita* mRNA and reflected by a decrease in MHC II staining in the E-cadherin–positive epithelial cells after rIL-22 treatment (Fig. 3, K and L). While we cannot exclude other effects of rIL-22 treatment, such as direct impact on APCs (Ke et al., 2011), we did not see any effect of IL-22 in suppressing IFN*γ*-induced MHC II on bone marrow–derived macrophages or THP-1 cells (human leukemia monocyte; Fig. S2, D and E). Overall, this suggests that IL-22 has multiple beneficial effects to minimize pathology in the mucosa and one of these could potentially be mediated by the downregulation of epithelial MHC II expression (Jamwal et al., 2020).

*Il22ra1*^*KO*^ animals were more susceptible to DSS-induced colitis, presenting with increased pathology, decreased body weight, substantial diarrhea, depletion of goblet cells, and increased colon weight/length ratio in these animals (Fig. 3, M–P; and Fig. S2 F). Moreover, we observed a marked increase in *Ciita* and a decrease in *Reg3γ*, an antimicrobial peptide, which was associated with a significantly increased epithelial cell–MHC II expression in the *Il22ra1*^*KO*^ animals (Fig. 3 F). WT intestinal organoids treated with IL-22–activated p-Stat3 and RNA sequencing (RNA-Seq) analyses confirmed that the ER stress response was markedly downregulated in the presence of IL-22 (Fig. S2, G and H). Administration of several long-circulating forms of IL-22 showed improved pathology in models of IBD, which has mostly been attributed to IL-22–driven epithelial cell renewal promoting mucosal barrier repair (Rothenberg et al., 2019; Tang et al., 2019). Some contrasting reports have challenged the rationale for exogenous IL-22 supplementation in patients with active colitis, showing that IL-22 may drive an ER stress response in the epithelial cells (Powell et al., 2020). However, our work suggests that IL-22 only transiently upregulates ER stress in the epithelial cells, which is associated with an increase in protein biosynthesis and differentiation of epithelial cells to a secretory phenotype (Moniruzzaman et al., 2019). Consistent with our hypothesis, specific deletion of IL-22RA1 on IECs using the Villin-Cre mice showed a substantial increase in epithelial MHC II levels (Fig. 3 Q). These data support the notion IL-22–mediated suppression of epithelial MHC II, potentially via ER stress, will be beneficial in the treatment of chronic inflammatory diseases such as IBD.

### IL-22**–**mediated suppression of epithelial MHC II expression is associated with increased mortality in acute infection

By halting protein synthesis, ER stress and the unfolded protein response is a key mechanism by which mucosal epithelial cells combat viral infections (Forsyth and Eisenlohr, 2016; Wang et al., 2018). A detrimental role for IL-10 has been reported in multiple infections, which in addition to its immunosuppressive function may also be due to its ability to suppress ER stress (Anderson et al., 2008; Brooks et al., 2006, 2008; Sun et al., 2010). There are mixed reports on the role of IL-22 in viral infection, which we would argue is most likely related to the timing of IL-22 exposure. While IL-22 promotes wound repair following influenza A infection, it enhances susceptibility to Zika virus (Liang et al., 2020; Pociask et al., 2013).

**Figure 4.**
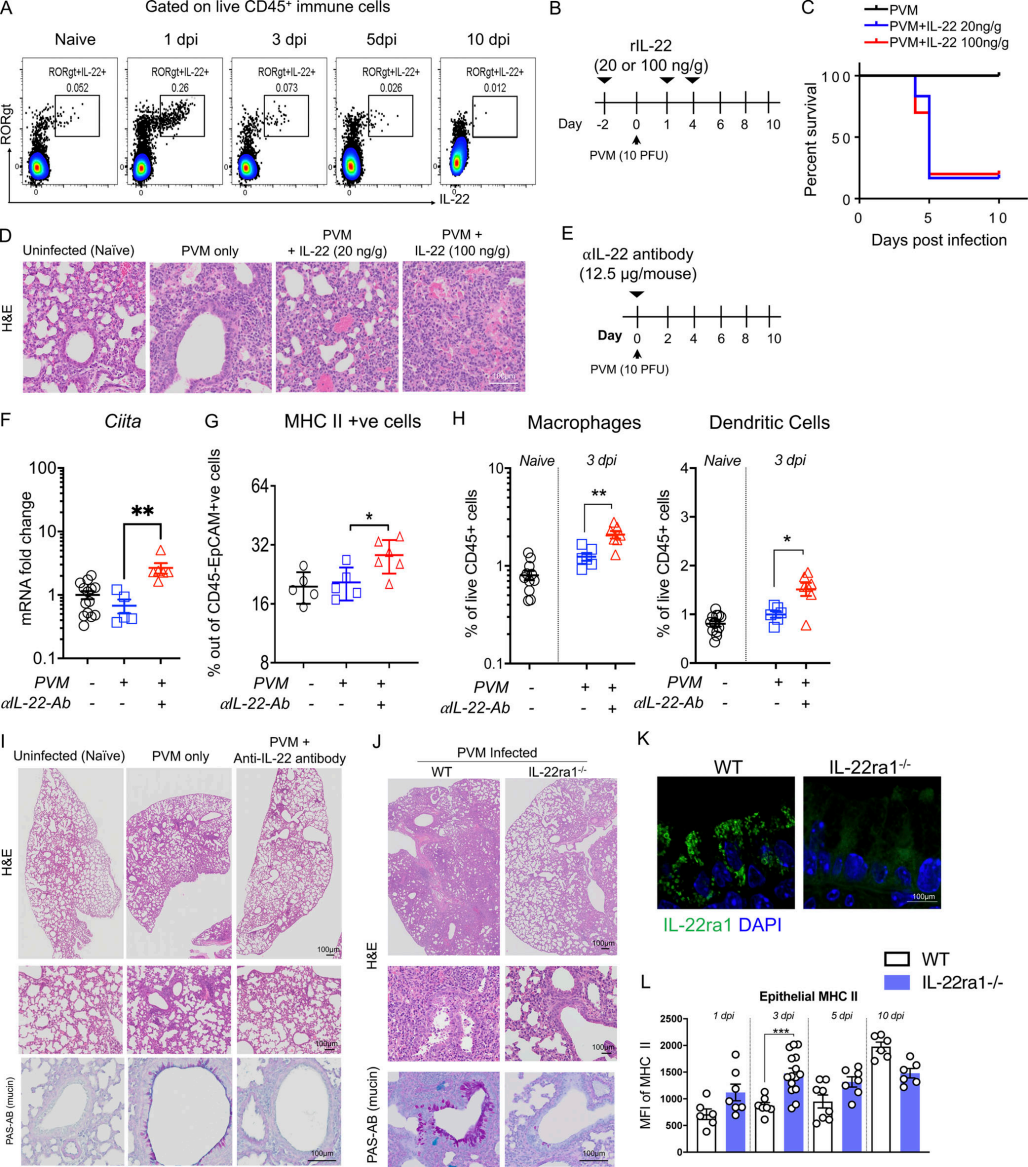
**IL-22 suppression of epithelial MHC II is associated with exacerbated pathology in acute respiratory viral infection. (A)** Flow cytometry plots showing abundance of RORγt^+^ IL-22^+^ respiratory tract immune cells after infection of C57BL/6 mice with 10 PFU PVM at indicated dpi. **(B)** Schematic experimental diagram of rIL-22 (20 or 100 ng/g) treatment prior to PVM infection. **(C)** Survival curves. **(D)** Representative H&E-stained lungs collected on day 10. **(E)** Schematic of experiment with PVM-infected C57BL/6 animals treated with α-IL22-antibody (12.5 μg/mouse) on the day of infection. **(F)** Respiratory tract mRNA expression of *Ciita* by qRT-PCR. **(G and H)** Flow cytometry determination of the relative frequency of MHC II^+^ and H macrophages and dendritic cells respiratory tract epithelial cells on day 3 after infection. **(I)** Representative H&E- and PAS-AB–stained respiratory tract sections. **(J)** Representative respiratory tract H&E- and PAS-AB–stained respiratory tract sections on day 10 of PVM-infected *Il22ra*^*WT*^ (Il-22ra1^fl/fl^; littermate controls) and *Il22ra1*^*KO*^ (CMV^cre^ × Il-22ra1^fl/fl^) mice. **(K)** Immunofluorescence staining demonstrating the lack of IL-22RA1 on epithelial cells from *Il22ra1*^*KO*^ animals. **(L)** MFI of MHC II expression on respiratory tract epithelial cells during the course of PVM infection in *Il22ra*^*WT*^ and *Il22ra1*^*KO*^ mice. Statistics: mean ± SEM (*n* = 5–12); data are representative of three independent experiments. One-way ANOVA, Bonferroni’s post hoc test; *P < 0.05, **P < 0.01, and ***P < 0.001.

**Figure S3.**
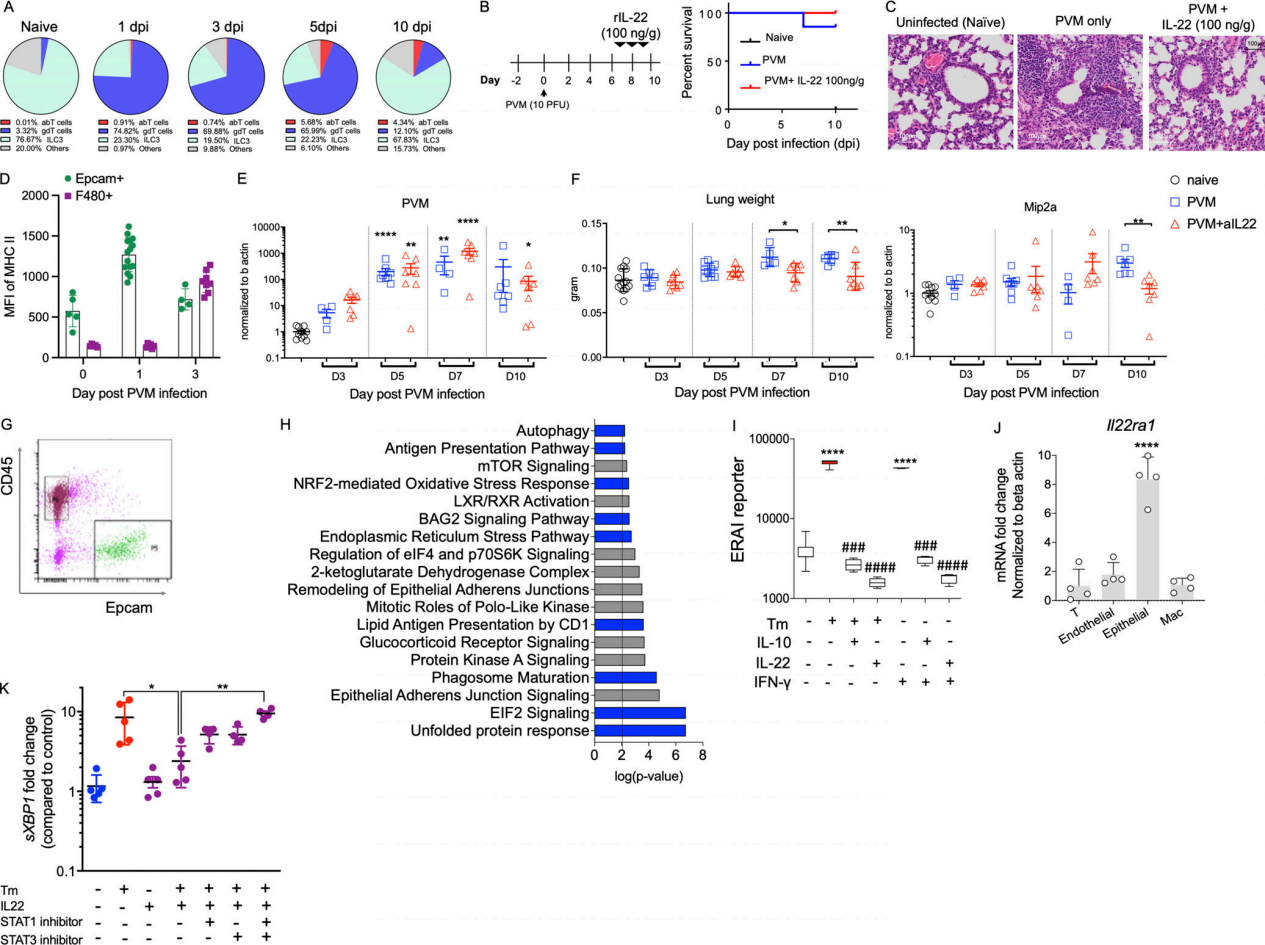
**Late IL-22–mediated suppression of epithelial MHC II does not alter pathology in viral infection. (A)** Source of IL-22 was assessed in naïve animals or animals with PVM infection; flow cytometric relative data presented as percentages. abT, αβ T cells; gdT, γδ T cells. **(B)** Schematic experimental diagram and survival curve. **(C)** Representative H&E staining images of the lungs of WT mice infected with PVM (100 PFU) at day 0 and treated with rIL-22 (100 ng/mouse) on days 6, 8, and 10 prior to analysis at day 10. **(D)** MFI of MHC II on Epcam^+ve^ cells or F4/80^+ve^ cells during PVM infection. **(E)** Relative expression of *PVM-sh* assessed by qRT-PCR. **(F)** Lung weight and chemokine, *Mip-2a*, gene expression level at indicated day in the α-IL22 antibody (12.5 μg/mouse) treated mice as in A. **(G)** Representative flow cytometric plot showing FACS-sorted Epcam cells for RNA-Seq analysis from WT animals infected with PVM (10 PFU) with and without α-IL22-antibody treatment, on day 3 after infection. **(H)** DAVID gene ontology analysis showing the upregulation of ER stress, antigen presentation and the unfolded protein response pathways in the EpCAM^+ve^ cells from animals treated with α-IL22-antibody. Data are presented as mean ± SEM (*n* = 6–7). **(I)** Fluorescence of ER stress activated indicator (ERAI) reporter transfected in LS174T cells treated with Tm (10 µg/ml), IL-10 (50 ng/ml), IL-22 (50 ng/ml), or IFNγ (50 ng/ml), alone or in combination for 24 h. **(J)** The relative expression level of *IL-22ra1* mRNA was measured in FACS-sorted epithelial cells (Epcam^+^), endothelial cells (CD31^+^), T cells (CD3^+^), and macrophages (Cd11b^+^) from naïve C57BL/6 mice. **(K)** HBECs treated with Tm (1 µg/ml), IL-22 (10 ng/ml) alone, or in combination in the presence of 50 µmol/liter of STAT1 and STAT3 inhibitors and *sXBP1* expression was assessed by qRT-PCR. Data are representative of two independent experiments and presented as mean ± SEM (*n* = 4–10). One-way ANOVA, Bonferroni’s post hoc test; *P < 0.05, **P < 0.01, and ****P < 0.0001. ^###^P < 0.001 and^ ####^P < 0.0001 compared to cellular stressors Tm and IFNγ.

IL-22–mediated suppression of MHC II expression is beneficial in chronic inflammation, but we hypothesized that activation of this pathway during infection would be detrimental. To explore this pathway, we inoculated mice with pneumonia virus of mice (PVM), the ortholog of respiratory syncytial virus (Sikder et al., 2023). IL-22 levels were endogenously upregulated in the animals at 1 d post infection (dpi). While the main source of endogenous IL-22 in the naïve lungs is innate lymphoid cells, with infection, RORγt^+^ T cells produce the majority of IL-22 (Fig. 4 A and Fig. S3 A). As endogenous IL-22 was upregulated at 1 dpi, to assess the impact of IL-22–induced suppression of MHC II with respect to increased susceptibility to viral infection, we administered rIL-22 2 d prior to PVM inoculation (Fig. 4 B). Of note, while the animals infected with PVM alone survived, 89% the animals administered with rIL-22, at either 20 or 100 ng/g per mouse, died at 5 dpi (Fig. 4 C). H&E demonstrated there was increased pathology in the animals treated with rIL-22, mirroring the increased mortality rate in these animals (Fig. 4 D). In stark contrast, when IL-22 was administered on days 7, 8, and 9 after infection, after the peak of infection, there was no effect on mortality or respiratory tract pathology (Fig. S3, B and C).

The increase in mortality observed when rIL-22 was administered prior to infection made it difficult to assess the role of IL-22–driven suppression of epithelial MHC II expression. Therefore, to ensure the epithelial cells remained intact, we instead blocked IL-22 during PVM infection. Endogenous IL-22 increased by 1 dpi, which may be a protective mechanism driven by the virus itself. This is again supported by viewing the biology of IL-10 as an example, where a range of viruses are reported to promote the production of IL-10 to impair antigen presentation capacity of APCs (Barthelemy et al., 2017; Rojas et al., 2017). Therefore, we administered anti-IL-22–neutralizing antibody on the day of infection (Fig. 4 E). On day 3, there was a significant upregulation of *Ciita* in PVM-infected animals treated with anti-IL-22 antibody (Fig. 4 F). Corroborating this data, we observed significant increases in the proportion of Epcam^+ve^ cells expressing MHC II (Fig. 4 G). However, concomitant with the increase in epithelial MHC II, there was an increase in the number of macrophages and dendritic cells in the respiratory tract in the PVM-infected treated with anti-IL-22 compared to the control group (Fig. 4 H). Further analyses demonstrated that MHC II cell surface abundance, as shown by increased MFI, was higher in Epcam^+ve^ cells on 1 dpi compared with F4/80 cells^+ve^ cells, showing again that epithelial MHC II expression is elevated prior to the increase in macrophage infiltration (Fig. S3 D). Importantly, no significant changes were observed in the viral abundance between PVM-infected animals and PVM-infected animals treated with anti-IL-22 (Fig. S3 E). The pathology was significantly improved in animals treated with anti-IL-22 at 10 dpi, with decreased immune cell infiltration and decreased mucin secretion (Fig. 4 I). Consistent with the histological appearance, there was a decrease in respiratory tract weight, which is a measure of gross inflammation, and a decrease in *Mip2a* at 10 dpi in the animals treated with anti-IL-22 (Fig. S3 F). This suggested that the change in kinetics in the absence of IL-22 and early increase in epithelial MHC II expression could potentially lead to the increase in APC infiltration and could be the mechanism resulting in the earlier resolution of pathology without altering viral load.

Next, we inoculated *Il22ra1*^*KO*^ mice lacking the IL-22RA1 on respiratory tract epithelial cells and infected with PVM (Fig. 4 K). *Il22ra1*^*KO*^ animals had enhanced respiratory tract epithelial MHC II expression at baseline (Fig. 2), and at 3 dpi, the *Il22ra1*^*KO*^ mice expressed significantly higher levels of epithelial MHC II (Fig. 4 L). In contrast, WT animals did not reach this level of epithelial MHC II expression until 10 dpi (Fig. 4 L). No changes in the viral load were observed in early infection (data not shown); however, *Il22ra1*^*KO*^ were protected from the PVM-induced pathology (Fig. 4 J). As the IL-22RA1 subunit is shared by IL-20 and IL-24 in addition to IL-22, the effects of these cytokines in modulating epithelial MHC II expression cannot be completely ruled out (Ouyang and Valdez, 2008). However, the neutralization of IL-22 alone led to a significant increase in epithelial MHC II expression, which correlated with the increase in APCs in the lung and improved pathology.

To confirm the mechanism by which IL-22 regulates epithelial MHC II expression, we isolated Epcam^+ve^ cells from PVM-infected animals or PVM-infected animals treated with anti-IL-22 (Fig. S3 G). RNA-Seq analyses on these epithelial cells highlighted that 9 of the 18 major pathways upregulated in the animals treated with anti-IL-22 were associated with ER stress and antigen presentation (highlighted in blue; Fig. S3 H). These experiments suggest that in the setting of an acute viral infection, early IL-22–mediated suppression of epithelial MHC II expression may be detrimental. By neutralization of IL-22 or by using the *Il22ra1*^*KO*^, we clearly observed a change in the kinetics epithelial MHC II expression (independent of viral load), demonstrating that early elevation of epithelial MHC II expression can potentially result in enhanced efficiency in the resolution of infection.

### IL-22 directly suppresses epithelial MHC II

**Figure 5.**
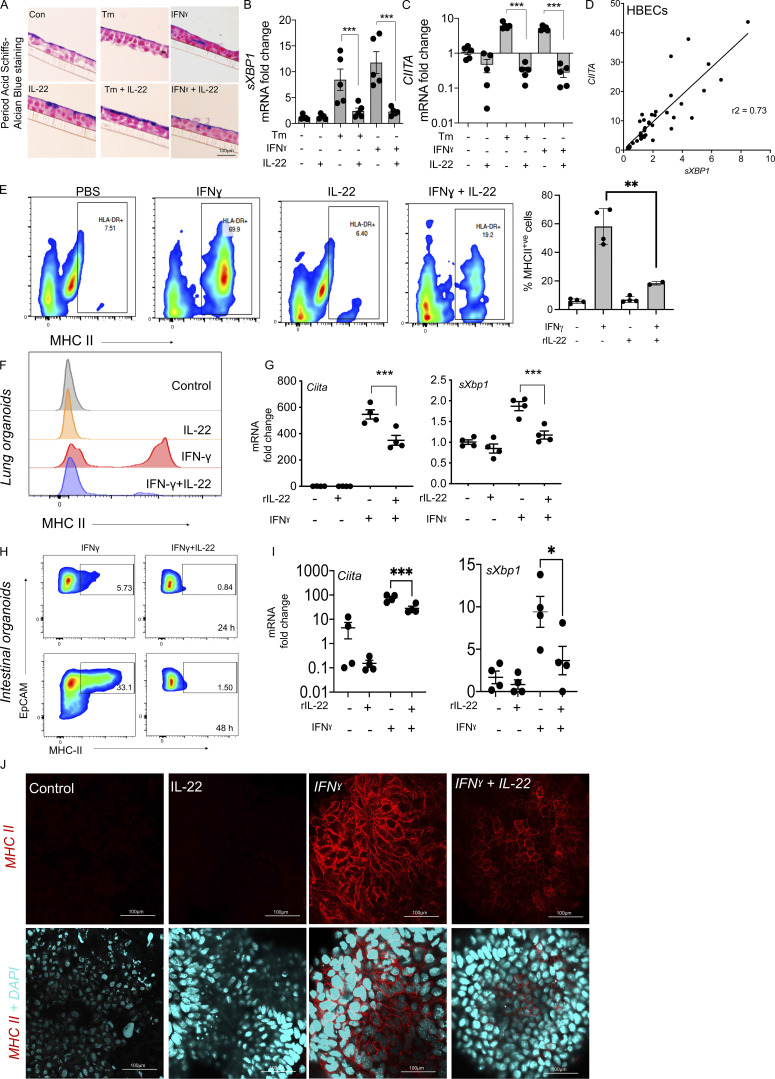
**IL-22 suppresses epithelial MHC II accompanied by a suppression of ER stress. (A)** Histological sections of monolayer cultures showing mucin production by HBECs (five donors) treated with Tm (1 µg/ml) or IFNγ (0.1 µg/ml) alone or in combination with IL-22 (10 ng/ml) for 24 h. **(B and C)** Relative mRNA expression of ER stress marker (*sXBP1*) and *CIITA* were measured by qRT-PCR in the HBECs treated as in A. **(D)** Correlation of *CIITA* and *sXBP1* expression by HBECs. **(E)** Representative flow cytometric plots and relative frequency of total MHC II expression in HBECs treated with IFNγ (0.1 µg/ml) with and without alone or in combination with IL-22 (10 ng/ml). **(F and G)** Flow cytometry histograms for cell surface MHC II (F) and (G) qRT-PCR for *Ciita* and *sXbp1* mRNA showing IL-22 (10 ng/ml; 24 h) mediated suppression of IFNγ (1 µg/ml)-induced MHC II and ER stress in cultured murine lung organoids from C57BL/6 mice. **(H–J) **Flow cytometry scatter plot of intestinal organoids from C57BL/6 mice treated with IFNγ (1 µg/ml) ± IL-22 (10 ng/ml) for 24 or 48 h (H), qRT-PCR of *Ciita* and *sXbp1* mRNA (I), and immunofluorescence staining for MHC II in intestinal organoids (J). Statistics: mean ± SEM (*n* = 4–12); data are representative of two independent experiments. One-way ANOVA, Bonferroni’s post hoc test; *P < 0.05, **P < 0.01, ***P < 0.001, and ****P < 0.0001.

To identify the direct IL-22–mediated effects on epithelial MHC II, we obtained primary human bronchial epithelial cells (HBECs) from five different healthy, non-smoking adult donors and differentiated these at air–liquid interface for 21 d to mirror the physiological situation, as this forms a monolayer with epithelial cells and goblet cells (Fig. 5 A). HBECs treated with ER stress inducers, Tunicamycin (Tm) or IFNγ, showed a significant increase in the ER stress marker *spliced-XBP1*, which was strongly correlated with the increase in MHC II regulator, *CIITA* (Fig. 5, B–D). This was confirmed using flow cytometry as IL-22 potently inhibited IFNγ-induced MHC II in HBECs (Fig. 5 E).

Mechanistically, we confirmed that both IL-10 and IL-22 suppressed IFNγ-induced ER stress in epithelial cells, but IL-22 was more potent than IL-10 (Fig. S3 I). Although IL-22 and IL-10 belong to the same sub-family of cytokines, what differentiates these two cytokines is the cellular expression of their cytokine-specific receptor subunits. IL-22RA1 is mainly expressed in epithelial cells and importantly is absent in immune cells (Fig. S3 J), whereas IL-10 is expressed by most leukocyte subtypes (Ouyang and Valdez, 2008). We proposed that IL-22–mediated suppression of MHC II (and *CIITA*) is linked to its potent ability to suppress ER stress (Gronke et al., 2019; Gulhane et al., 2016; Hasnain et al., 2014; Lindemans et al., 2015; Moniruzzaman et al., 2019). We found that IL-22 suppressed ER stress induced by Tm or IFNγ, with an approximately fivefold decrease in *spliced-XBP1* (Fig. 5 B), which was dependent on both STAT1 and STAT3 (Fig. S3 K). There was a strong correlation between IL-22 suppression of *sXBP1* and *CIITA*/MHC II (Fig. 5, B–E). Taken together, these results indicated that IL-22 suppresses epithelial MHC II expression, potentially by reducing trafficking to the cell surface, which is reminiscent of the effects of the related cytokine, IL-10, in suppressing MHC II expression on professional APCs (Koppelman et al., 1997).

We next isolated the direct effects of IL-22 on epithelial cells by using prolonged cultured primary intestinal and lung organoids. IL-22 alone did not alter the very low levels of cell surface MHC II or MHC II mRNA expression in either intestinal or lung organoids (Biton et al., 2018). However, IL-22 cotreatment with IFNγ prevented IFNγ-mediated upregulation of epithelial MHC II expression on the cell surface (Fig. 5, F and H). Quantitative RT-PCR (qRT-PCR) analyses confirmed that IFNγ-induced *Ciita* expression in respiratory and intestinal organoids was accompanied by an increase in ER stress (*sXbp1*; Fig. 5, G and I). Importantly, both IFNγ-induced *Ciita* and *sXbp1* were significantly reduced by IL-22, and this is reflected in the immunofluorescence staining of MHC II on intestinal organoids (Fig. 5 J).

These results are consistent with our hypothesis that IL-22–mediated suppression of epithelial MHC II expression via the suppression of ER stress contributes to immune tolerance at the mucosal surfaces. Taken together, our study provides a new explanation for the multifaceted reports of IL-22 acting as an anti- or pro-inflammatory cytokine, demonstrating for the first time a direct role of IL-22 in modulating the immune response. IL-22–driven suppression of epithelial MHC II may break the cycle of inflammation during chronic inflammatory diseases. While this supports the use of IL-22 in chronic inflammatory conditions such as IBD, it is possible that epithelial MHC II suppression during acute infection delays the initiation of an appropriate immune response, resulting in increased pathogen-induced pathology. With several long-circulating forms of IL-22 in clinical trials for IBD, it will be prudent to evaluate the effects of IL-22 treatment on susceptibility to infection. Beyond the mucosa, we suggest that IL-22–mediated suppression of MHC II expression may have relevance to other sites where IL-22 receptor is highly expressed.

## Materials and methods

### Mice

All mouse work was conducted in accordance with ethics approved by the University of Queensland Animal Experimentation Ethics Committee. WT, C57BL/6 mice (Australian Resource Centre), *Winnie*, *Il22ra1*^*KO*^, and littermate controls on a C57BL/6 background (bred in-house) were housed under SPF conditions. GF C57BL/6 animals housed in the Trexler-type soft-sided isolators. All animals were kept in the Biological Research Facility at the Translational Research Institute (TRI), free of *Norovirus* and *Helicobacter*. Mixed-gender neonates at day 7 were infected with pneumovirus intranasally (PFU defined within experiments). PVM-infected animals were treated with recombinant IL-22 (20 or 100 ng/g) or anti-IL-22 antibody (12.5 µg/mouse), and schematics show the treatment regimens. 5–6-wk-old male C57BL/6 animals were challenged with 2.5% DSS in drinking water and subsequently treated with recombinant IL-22 (100 ng/g of body weight, intraperitoneally). 5–6-wk-old male *Winnie* animals were treated with recombinant IL-22 (100 ng/g of body weight, intraperitoneally). 5–6-wk-old mixed gender *Il22ra1*^*KO*^ (CMV^-Cre^ × IL-22RA^fl/fl^) and their littermate controls *Il22ra1*^*WT*^ (IL-22RA^fl/fl^) animals were challenged with 1.5% DSS (drinking water) or neonates were infected with PVM (intranasally). GF animals, mixed gender, were tested at 6–8 wk. Mice with colitis were daily monitored and scored for their body weight change, rectal bleeding, and stool consistency (0 = hard, 1 = soft but forms in a shape, 2 = soft but forms in a shape and falls apart when picked up, 3 = no form, 4 = watery).

### HBECs

The study was approved by the Mater Human Research Ethics Committee, Brisbane, Australia (HREC/14/MHS/26). Patient consent was received, after which bronchial brushings were obtained from five non-smoking healthy control subjects with no evidence of respiratory disease, no history of use of any bronchoactive medications, and normal spirometry (forced expiratory volume [FEV1], forced vital capacity [FVC], and FEV1:FVC ratio all lying within the normal range). The samples were provided through the David Serisier Respiratory Biobank. Briefly, brushings were expanded in bronchial epithelial growth medium and seeded onto 0.03 mg/ml collagen-coated transwells when they reached 80% confluence. As per the schematic in Fig. 2, cells were introduced to air–liquid interface by media removal after 7 d on transwells. Differentiation media supplemented basally with BPE high protein, insulin, hydrocortisone, GA-1000, transferrin, epinephrine, human epidermal growth factor, inducer from SingleQuot kit, and 50 nM fresh retinoic acid. Differentiation media was replenished every second day for 20 d. Day 21 cells were treated with low-dose Tm (1 µg/ml), IFNγ (0.1 µg/ml), and/or IL-22 (10 ng/ml). To determine the pathway activated after IL-22 treatment, HBECs were treated with Tm, IL-22 as above in the presence of 50 µmol/liter of STAT1 (fludarabine) and STAT3 (VI S31-201) inhibitors for 24 h. Cells were collected using lysis buffer for RNA extraction and transwells were fixed in 4% paraformaldehyde, embedded, and sectioned for H&E staining.

### Cell lines

LS174 T cells were transfected with the ERAI reporter (F-XBP1ΔDBD-venus) as we have described before (Hasnain et al., 2013). Cells were subsequently treated with Tm (10 µg/ml), IL-10 (50 ng/ml), IL-22 (50 ng/ml), or IFNγ (50 ng/ml) alone or in combination for 24 h. Splicing of XBP1 messenger RNA by IRE1α results in the translation of Venus-GFP but not an active form of XBP1, which is then detected using a POLARstar Omega plate reader. 16HBE cells were treated with 10 ng/ml IFNγ alone or ER stress inhibitors (either 35 μM/ml 4μ8c, or 2.5 mM/ml 4PBA) in combination with 10 ng/ml IFNγ for 24 h and then harvested with EDTA 10 mM and stained for MHC II detection via flow cytometry. THP1 cells were treated with cytokines for 24 h, either 10 ng/ml IFNγ, 100 ng/ml IL-22, or a co-treatment of both simultaneously. 3-(4,5-dimethylthiazol-2-yl)-2,5-diphenyltetrazolium bromidefor assay was used to assess cell viability throughout all the experiments and no changes were observed with treatment.

### Bone marrow**–**derived macrophage (BMDM) cultures

BMDMs were isolated from 8-wk-old WT mice in the presence of 50 ng/ml m-CSF for 6 d prior to treatment with either IFN-g (0.5 ng/ml) and/or IL-22 (100 ng/ml) for 24 h. Flow cytometry was used to assess the levels of CD11b^+^MHCII^+^ cells from the total live CD45^+^ cells from three replicates.

### Murine organoid cultures

Primary mouse epithelial cells were isolated from the intestine and respiratory tract using the Stappenbeck method as we have previously described (Gulhane et al., 2016; Miyoshi and Stappenbeck, 2013; Wang et al., 2021). Briefly, mouse intestine was cut longitudinally and mouse respiratory tract was washed with ice-cold PBS containing 1% pen-strep. Tissue was then cut in size of approximately a centimeter and treated with ice-cold 8 mM EDTA/PBS containing 1% pen-strep for 1 h at 4°C. Subsequently, tissue was incubated with 2 mg/ml collagenase and 50 μg/ml gentamycin in F12-DMEM containing 10% FBS, 1% Glutamax, and 1% pen-strep (washing medium) for 5–10 min at 37°C. Stem cells were then isolated using 10 ml medium through vortexing and centrifugation at 500 rpm for 5 min at 4°C. Cells were plated in basement membrane embedding (Miyoshi and Stappenbeck, 2013) in a 1:1 ratio and cultured in 50% L-WRN conditioned medium (a kind gift from Stappenbeck Lab, Washington University, St. Louis, MO, USA) together with Y27632 at 10 µM (Rho-associated protein kinase inhibitor) and SB431542 at 10 µM (the transforming growth factor-β type I receptor inhibitor). Organoids treated with PBS or IL-22 (100 ng/ml for 60 min) were harvested in radioimmunoprecipitation assay buffer with phosphatase and protease inhibitors for Western blotting. Protein concentrations in the lysates were determined using bicinchoninic acid assay kit (Thermo Fisher Scientific) and samples were stored at −80°C until use. 40–45 μg of proteins from each sample were resolved using NuPAGE 4–12% Bis-Tris protein gels (Invitrogen) at 100 V and transferred to a poly(vinylidene fluoride) membrane using a Thermo Fisher Scientific iBlot 2 dry blotting system. The membranes were then blocked in PBS Odyssey buffer for 2 h. Following incubation with primary antibody overnight at 4°C, membranes were washed with PBS-Tween and treated with fluorophore-conjugated secondary antibody for 2 h at room temperature. After washing the membranes, images were scanned using the Odyssey Imaging System and processed, and densitometric analysis was conducted on blots with positive bands using Image Studio Lite software (LI-COR Biosciences). Organoids were also kept in TRIzol for RNA isolation and qRT-PCR analyses or RNA-Seq.

### RNA-Seq

We performed next-generation sequencing in undifferentiated primary murine IECs treated with 100 ng/ml of IL-22 (4 h) and Epcam^+ve^ cell isolated from mice infected with PVM and PVM-infected animals treated with anti-IL-22 antibody. The mRNA sequencing was done by the Australian Genome Research Facility using HiSeq 2500 machine (Illumina) with a maximal read length of 100 bp. The differential gene expression was analyzed through edgeR (version 3.22.3) using R (The R Foundation; version 3.5.0). Raw gene counts per million were used for transcriptional comparison. The KEGG pathway and gene ontology analyses were done using David to identify the differentially expressed genes. Data are available through the National Center for Biotechnology Information under accession no. PRJNA1000210.

The qRT-PCR was performed according to the protocol described previously (Hasnain et al., 2014). Briefly, after desired samples were lysed using TRIzol (Invitrogen), pure RNA was isolated using ISOLATE II RNA Mini Kit from Bioline (Alexandria). For the animal samples, tissues were homogenized using beads (Lysing Matrix D Bulk; MP Biomedicals) in TRIzol and then followed the kit instructions. Equal 1 μg of RNA was then used to synthesize corresponding cDNA using a Bioline cDNA synthesis kit. Depending on the targeted genes, the cDNA was diluted up to 1:10 ratio to perform PCR. 2.5 μl of diluted cDNA, 0.75 μl of desired primer (Table S1), 3.75 μl of SYBR green (SensiFAST SYBR Lo-ROX kit; Bioline), and 0.5 μl of DNase and RNase free water were mixed together and run in a Real-Time PCR System (Applied Biosystems ViiA 7; Life Technologies Corporation) for 40 cycles. The Ct values were then analyzed using a ViiA 7 software (Life Technologies Corporation). The relative quantitation was determined by the ΔΔCt method and normalized to housekeeping gene *Tata/TATA box* and expressed as a fold difference to the mean of the relevant control samples. The amplification of the targeted genes was verified from the melting curve obtained from the experiment. The fold changes were analyzed and plotted using GraphPad Prism software (version 8).

### Histological analysis

Tissues were fixed in 4% formalin and subsequently embedded in paraffin and sectioned as per requirements. The tissue was sectioned at 5 μm and stained with H&E, PAS-AB, and viewed in a digital microscope (Olympus). Pathology in DSS-treated mice was blindly scored for each animal using H&E-stained slides according to the conditions such as (i) inflammation severity (0 = none, 1 = mild, 2 = moderate, 3 = severe), (ii) infiltration extent (0 = no infiltrate, 1 = infiltrate around crypt base, 2 = infiltrate reaching to muscularis mucosae, 3 = extensive infiltration reaching the muscularis mucosae and thickening of the mucosa with abundant edema, 4 = infiltration of the submucosa), (iii) epithelial damage (0 = normal morphology, 1 = some loss of goblet cells/some crypt abscesses or damage, 2 = loss of goblet cells in large areas/extensive crypt abscesses or damage, 3 = loss of crypts <5 crypt widths, 4 = loss of crypts >5 crypt widths, <20% ulceration, 5 = >20% ulceration), and (iv) percentage of epithelial damage—crypt abscessed, crypt loss, or ulceration (0 = 0%, 1 = 1–25%, 2 = 26–50%, 3 = 51–75%, 4 = 76–100%). To check mucins and the goblet cells, the sections were stained with AB and PAS reagents.

### Flow cytometry analysis

Cells from the respiratory tract and intestine were collected. Mesenteric lymph nodes were crushed through a 70-μm strainer and suspended in DMEM containing 10% FBS, 1% glutamax, and 1% pen-strep in ice. The single cells were collected through centrifugation at 500 rpm for 5 min at 4°C and resuspended in magnetic activated cell sorting buffer (Miltenyi Biotech). For cell-surface antigen staining, the samples were incubated with antibodies in magnetic activated cell sorting buffer for 30 min at 4°C, washed, and resuspended in 3% FBS. Following fixation, cells were permeabilized and incubated with antibodies overnight before being analyzed and identified through Fortessa (BD Biosciences) and FlowJo (Ashland). Epcam^+ve^ (epithelial), CD3^+^ (T cells), Cd11b^+^ (macrophages), and Cd31^+^ (endothelial) cells were sorted live, using FACSAria III cell sorter, and only samples with >98% efficiency were used. Sorted cells were collected in lysis buffer for qRT-PCR analyses.

### Statistical analysis

Data are presented as the mean ± SEM. The GraphPad Prism software program was employed for the analyses and plotting of the data. Nonparametric *t* test, one-way ANOVA followed by Dunnett’s post hoc test, or two-way ANOVA followed by Bonferroni’s post hoc test were performed wherever applicable to determine statistical differences as indicated in the figure legends.

### Online supplemental material

Fig. S1 shows the effects of IFNγ-induced MHC II on primary organoids and immune profiling of *Il22ra1*^*KO*^. Fig. S2 shows the effects of IL-22 treatment in the colitis models and the effects of IL-22 in suppressing IFNγ-induced MHC II in immune cells. Fig. S3 shows the effects of IL-22 exogenous treatment in the PVM model and the lung. Table S1 lists primers used in the study.

## Supplementary Material

Table S1 is a list of primers.

SourceData FS2 is the source file for Fig. S2.

## Data Availability

RNA-Seq data are available from the National Center for Biotechnology Information under accession no. PRJNA1000210. Requests for any other data should be directed to and will be fulfilled by the corresponding author.
