# Supplementary material for: Interleukin-22 suppresses major histocompatibility complex II in mucosal epithelial cells
Source: J Exp Med. 2023 Sep 11;220(11):e20230106. doi: 10.1084/jem.20230106 (PMC10494524; doi:10.1084/jem.20230106)
Supplement: Table S1 — is a list of primers. [file JEM_20230106_TableS1.docx]

Supplementary table 1: List of primers

| **Primer** | **Sequence** |
| --- | --- |
| HUMAN | |
| Human sXBP1 | Forward: GAGTCCGCAGCAGGTGC  Reverse: GAGTCCGCAGCAGGTGC |
| Human CIITA (F1, FIII and F-IV isoforms) | Forward: CTGTGTCTTCCGAGGAACTTC  Reverse: AGTTCACCATCGAGCCTTTC |
| Human *β-actin* | Forward: CCTGTACGCCAACACAGTGC  Reverse: ATACTCCTGCTTGCTGATCC |
| MOUSE |  |
| m*β-actin* | Forward: GAAATCGTGCGTGACATCAAA  Reverse: CACAGGATTCCATACCCAAGA |
| m*Tata box* | Forward: CTCAGTTACAGGTGGCAGCA  Reverse: ACCAACAATCACCAACAGCA |
| *mCiita*  (F1, FIII and F-IV isoforms) | Forward: TGCGTGTGATGGATGTCCAG  Reverse: CCAAAGGGGATAGTGGGTGTC |
| m*Grp78* | Forward: TGCTGCTAGGCCTGCTCCGA  Reverse: CGACCACCGTGCCCACATCC |
| m*sXbp1* | Forward: GAGTCCGCAGCAGGTGC  Reverse: CAAAAGGATATCAGACTCAGAATCTGAA |
| m*Mip2α* | Forward: ACGTGTTCCAGGACACAACA  Reverse: ACAAACCCTCCCCACCTAAC |
| *Reg3β* | Forward: ACTCCCTGAAGAATATACCCTCC  Reverse: CGCTATTGAGCACAGATACGAG |
| *Reg3γ* | Forward: GGCCATATCTGCATCATACCAG  Reverse: ATGCTTCCCCGTATAACCATCA |
| *PVM sh* | Forward: GCCTGCATCAACACAGTGTGT  Reverse: GCCTGATGTGGCAGTGCTT |
| *Il22ra1* | Forward: CCGAGGAGTCAGTGCTAAGG  Reverse: CATGTAGGGCTGGAACCTGT |
