# Supplementary material for: Interleukin-22 suppresses major histocompatibility complex II in mucosal epithelial cells
Source: J Exp Med. 2023 Sep 11;220(11):e20230106. doi: 10.1084/jem.20230106 (PMC10494524; doi:10.1084/jem.20230106)

Source Data
Supplementary Figure
2G

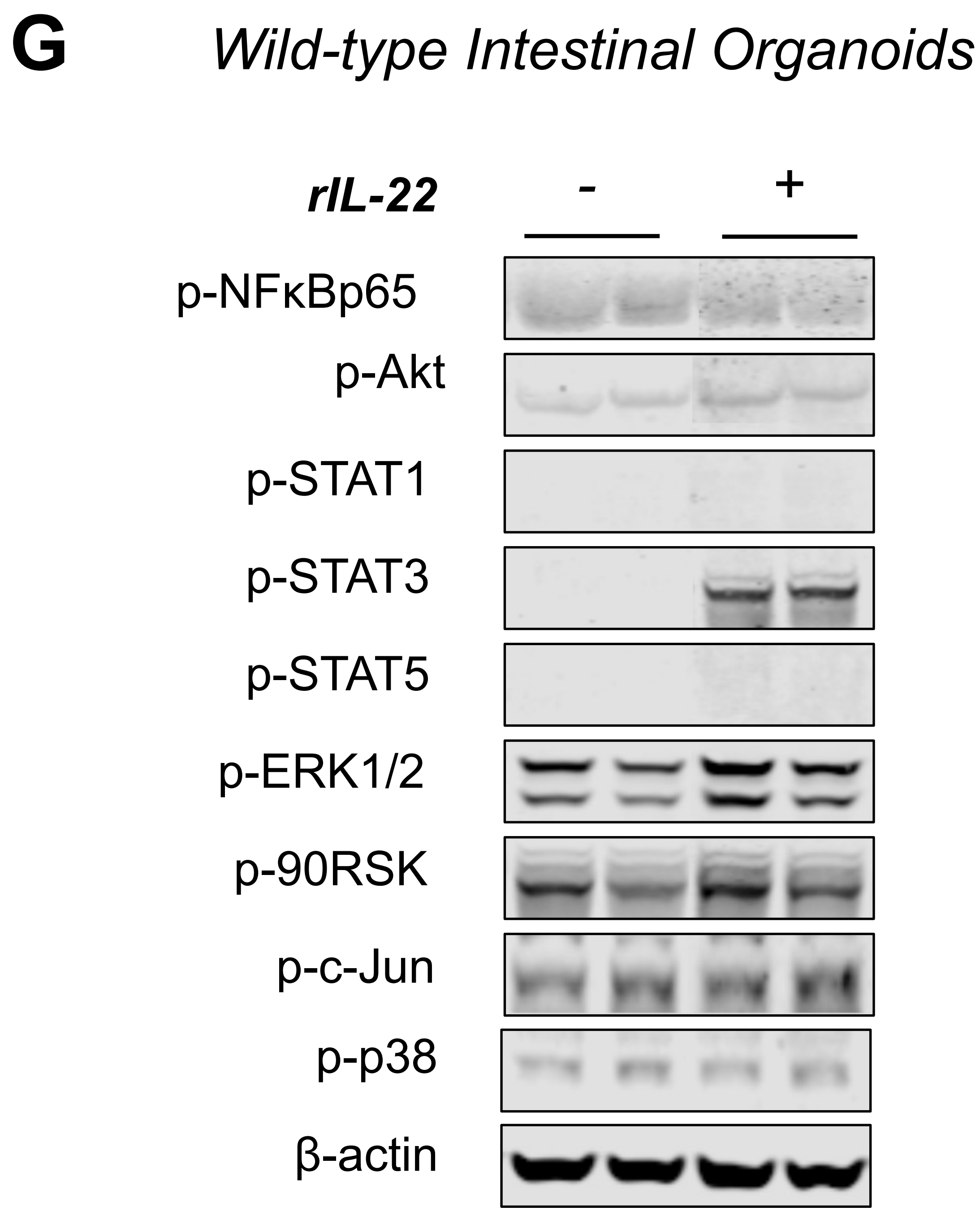

p-STAT5, p-NFkBp65, p-p38

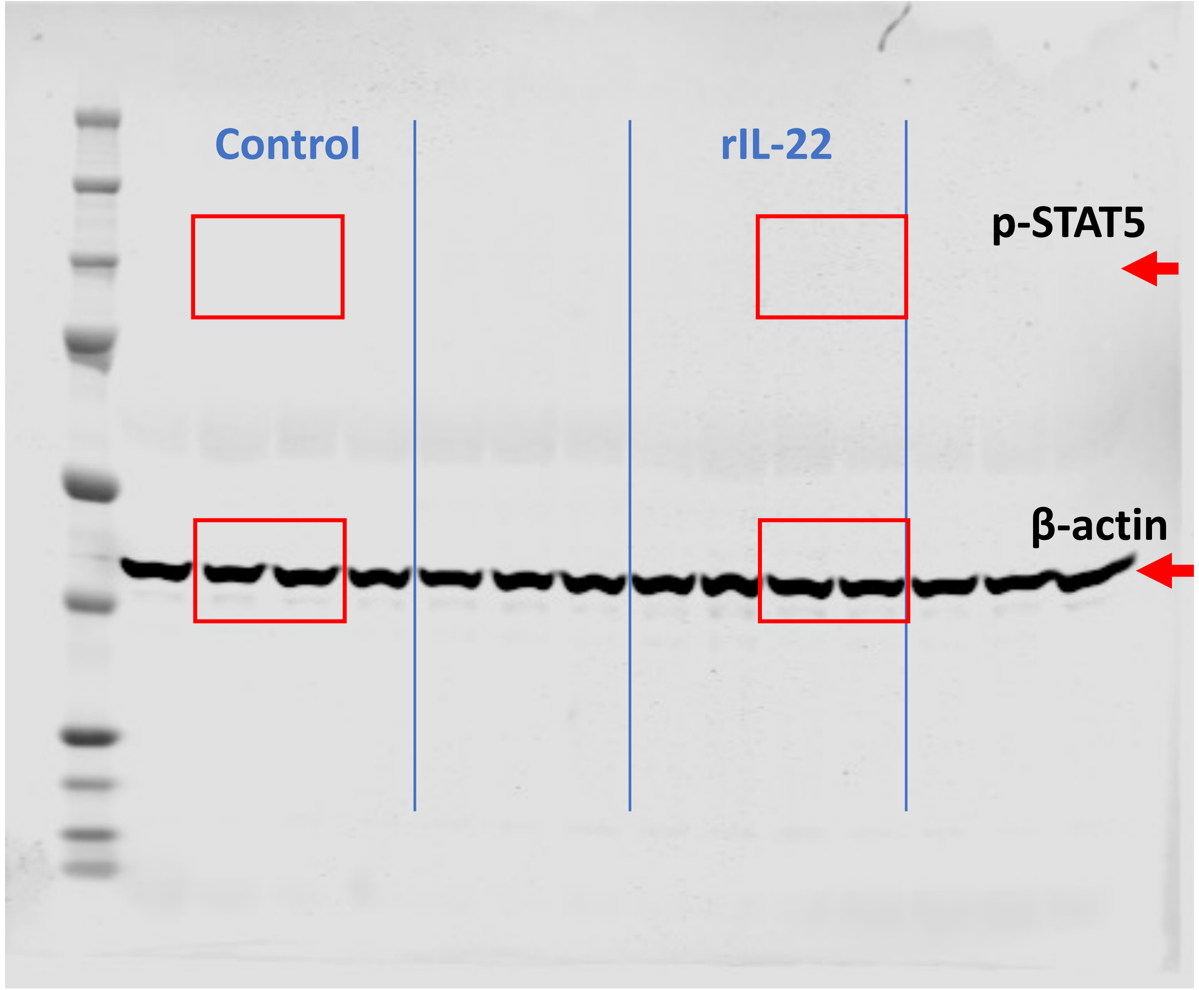

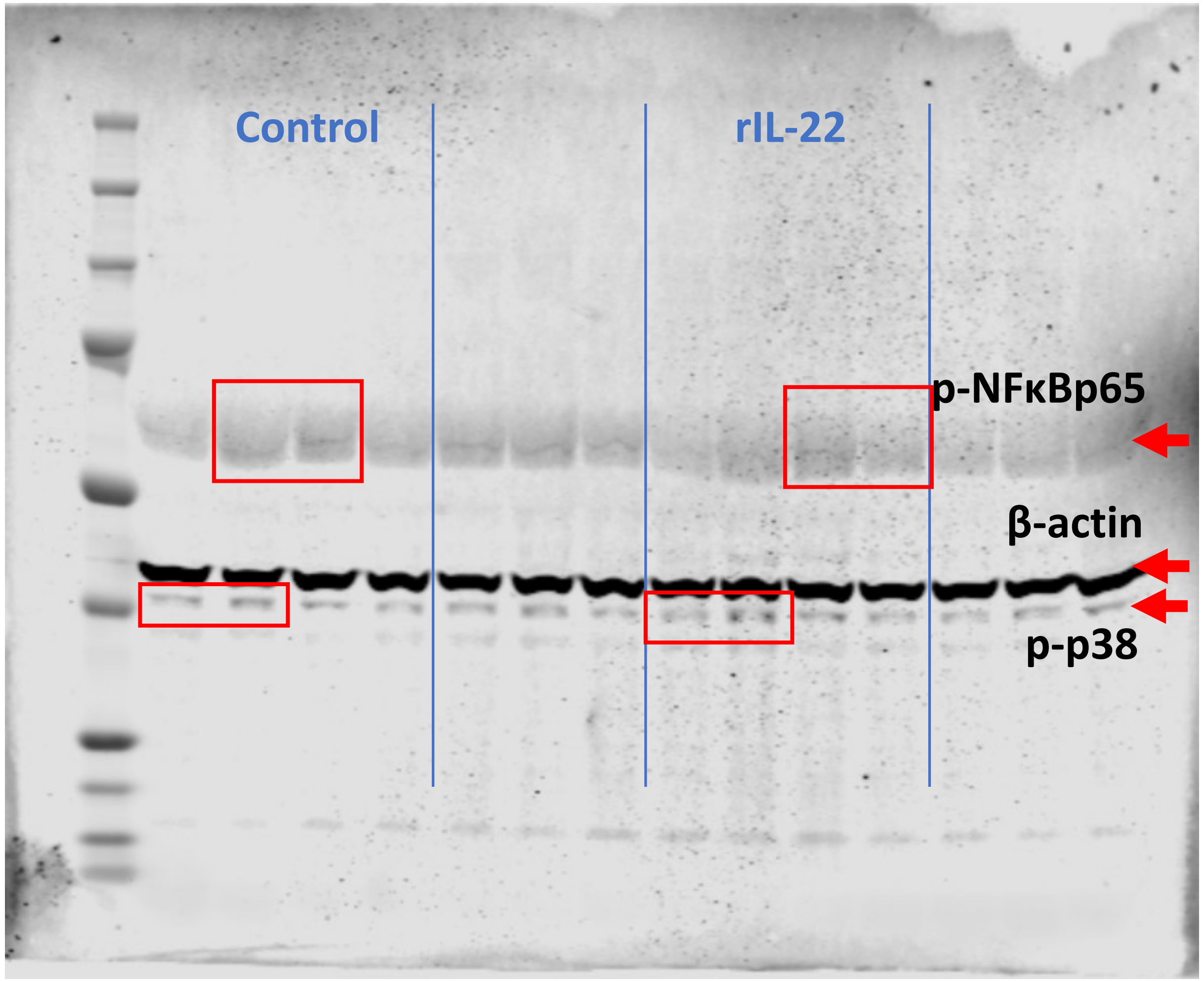

p-90RSK, p-c-jun

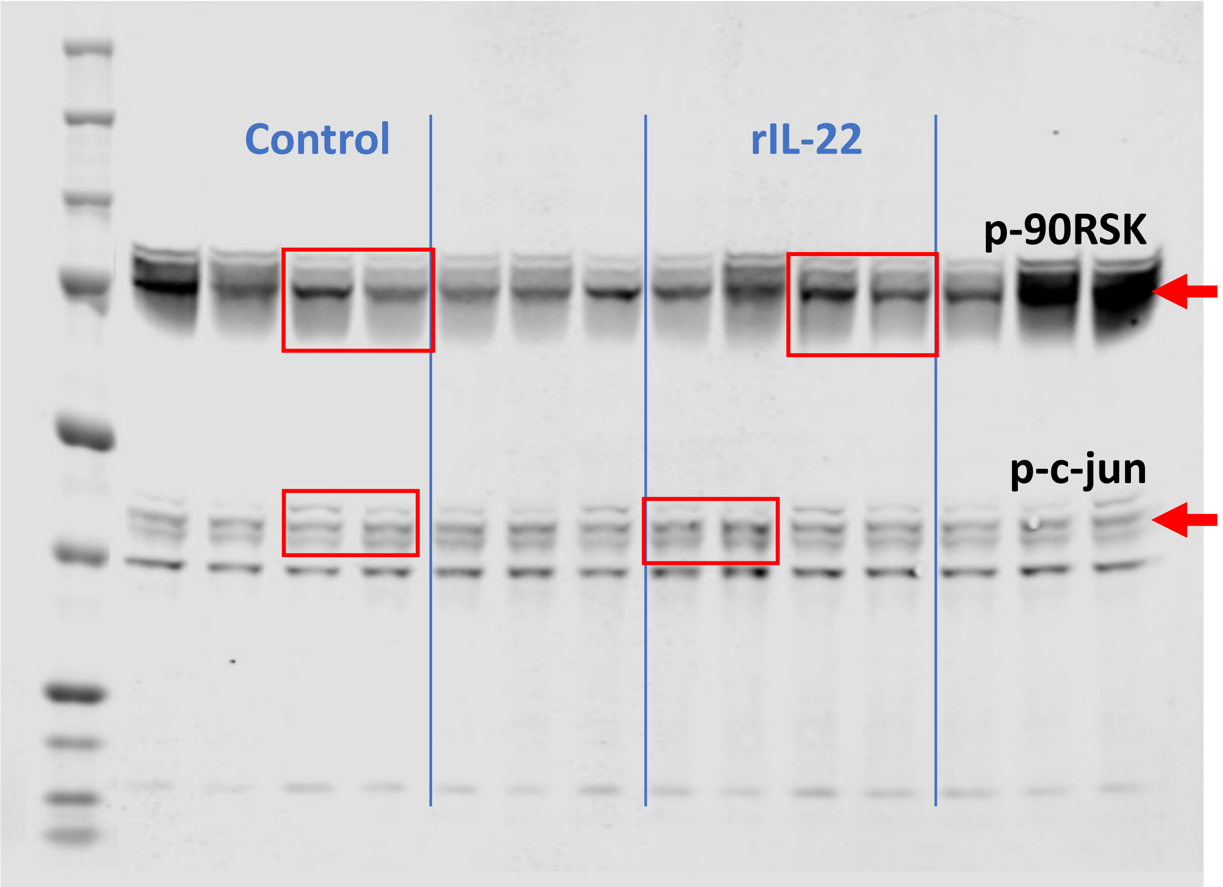

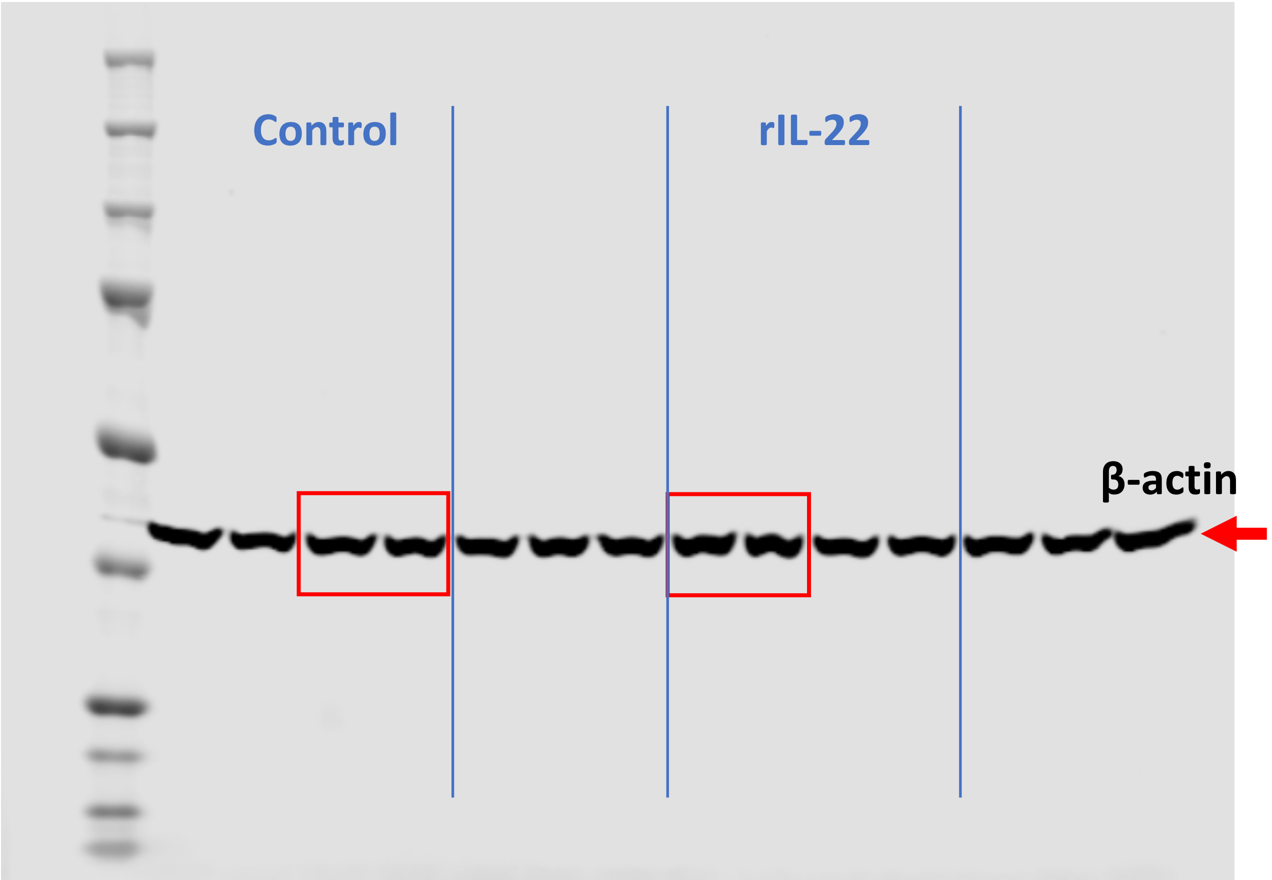

p-STAT1

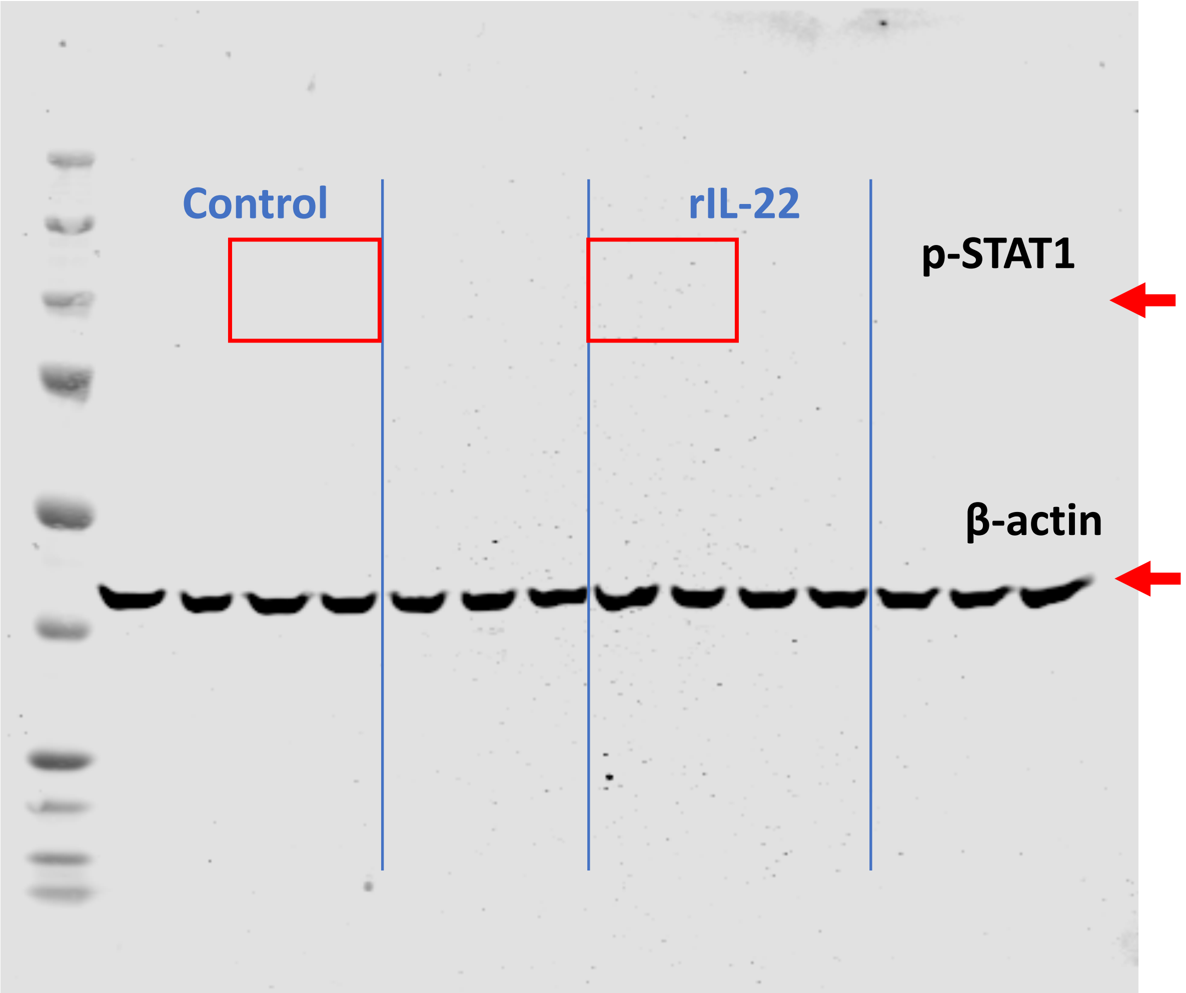

p-STAT3, p-Akt, p-ERK

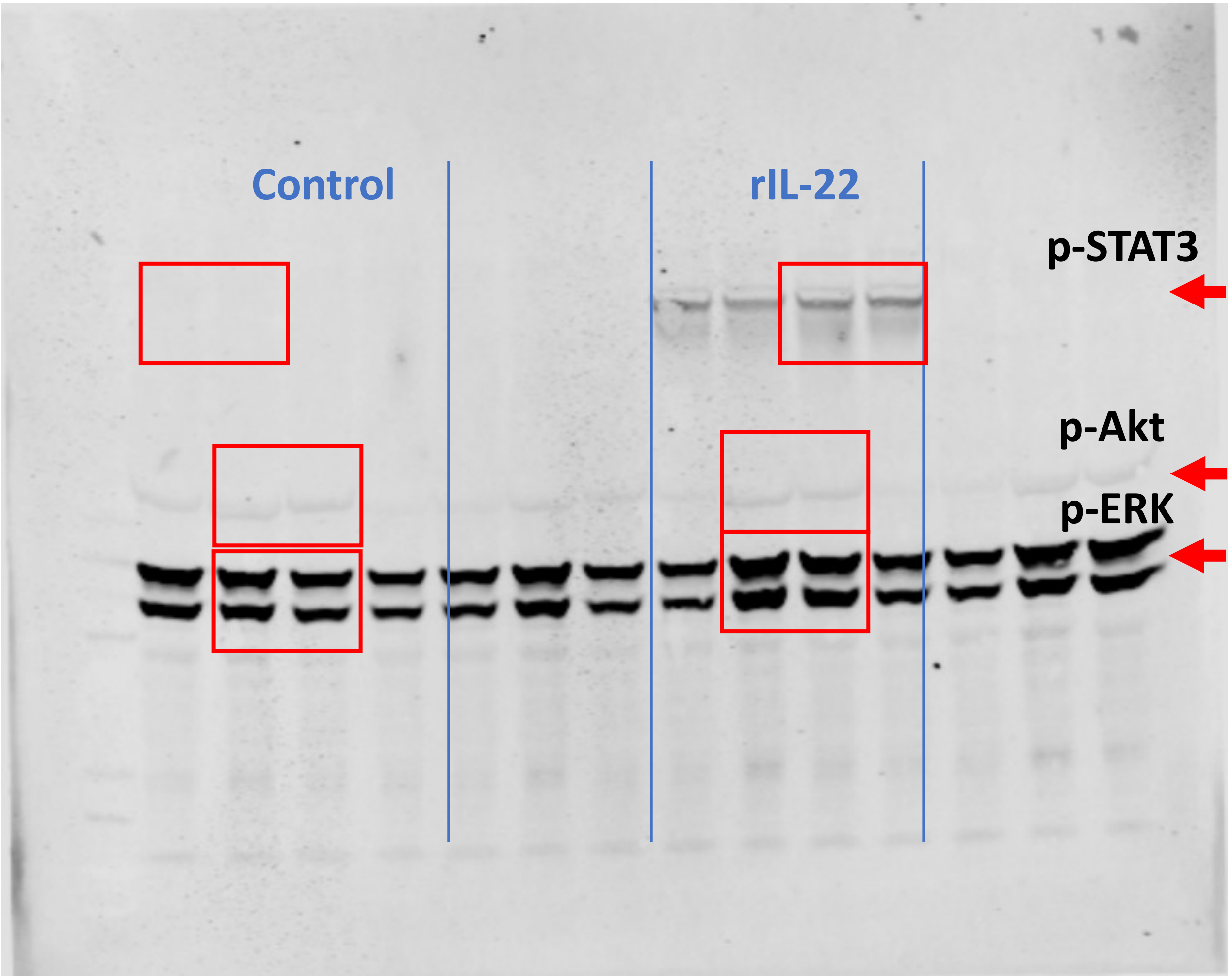

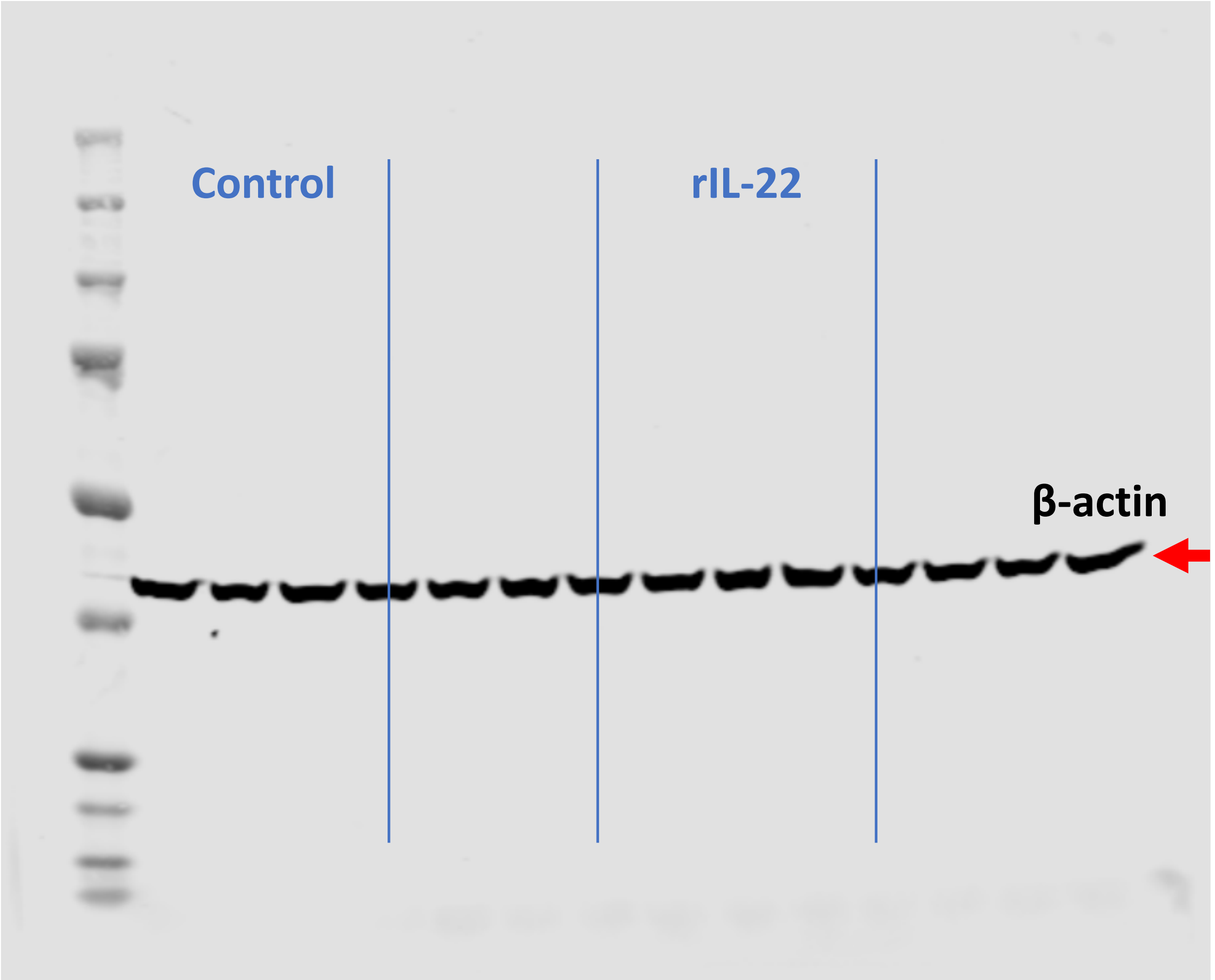

Supplement: SourceData FS2 — is the source file for Fig. S2. [file JEM_20230106_SourceDataFS2.pdf]
